# Prescribing a dose of transparency: a qualitative evaluation of AI explanations with cardiovascular healthcare professionals

**DOI:** 10.3389/frai.2026.1830201

**Published:** 2026-06-29

**Authors:** Sophie Haas, Malte Högemann, Oliver Thomas

**Affiliations:** 1 Information Management and Information Systems, University of Osnabrueck, Osnabrueck, Germany; 2 Smart Enterprise Engineering, German Research Center for Artificial Intelligence, Osnabrueck, Germany

**Keywords:** clinical decision support system, digital health, explainability, explanation method, human-centered evaluation, trustworthy AI

## Abstract

**Introduction:**

Chronic diseases, particularly heart failure, are associated with high mortality rates and frequent hospital readmissions, thereby placing a significant burden on healthcare systems and patients. Recent advancements in AI-supported telemonitoring offer a means to address these challenges through the early detection of deterioration. Yet, the black-box nature of AI can undermine physicians’ trust, underscoring the importance of explainable AI (XAI).

**Methods:**

This exploratory study examines clinicians’ perceptions of XAI in a prototype early-warning scenario for imminent heart failure decompensation based on tabular laboratory data. Three exemplary explanations are compared in interviews with 15 German cardiovascular healthcare professionals using the explanation satisfaction scale: SHapley Additive Explanations (SHAP), Counterfactual Explanations (CFEs), and Anchors.

**Results:**

SHAP was widely preferred for its intuitive graphical representation. CFEs were valued as an action-oriented method although their suitability for deriving therapeutic steps requires careful constraint and interpretation. Meanwhile, Anchors were considered too superficial.

**Discussion:**

Our findings suggest that no single explanation method fully meets clinicians’ needs. Instead, layered explanation designs that combine quick overviews with optional deeper insights may better support trustworthy AI use in early-warning scenarios based on tabular laboratory data.

## 1 Introduction

With the continuous enhancement of technological capabilities, the integration of artificial intelligence (AI) into our daily lives is becoming more and more pervasive. In healthcare, AI technologies are increasingly acknowledged for their capacity to support healthcare professionals in making informed clinical decisions by automating complex and time-consuming tasks (Bernal and Mazo, 2022). They hold the potential to enhance processes such as timely risk prediction, precise diagnosis, optimized treatment planning, and continuous patient monitoring, alongside the prospect of reduced costs (Bernal and Mazo, 2022; Abbas et al., 2025; Gambetti et al., 2025).

Despite these advances, a certain degree of caution is still warranted when integrating AI into daily clinical practice. The healthcare sector constitutes a high-stakes environment in which clinical errors resulting from flawed or misunderstood AI recommendations may have life-threatening consequences (Petch et al., 2022; Abbas et al., 2025; Gambetti et al., 2025). Yet, many of the most sophisticated AI models in healthcare operate as “black boxes,” so complex that their inner workings are inscrutable even to technical experts (Bernal and Mazo, 2022; Bergomi et al., 2025). While these models often achieve outstanding predictive performance, they typically do so at the expense of transparency (Bernal and Mazo, 2022; Senoner et al., 2024). The lack of explanations for their automated decisions is regarded as one of the primary barriers to their adoption in clinical practice (Markus et al., 2021; Petch et al., 2022; Abbas et al., 2025; Gambetti et al., 2025). Healthcare professionals must be able to understand and verify the logic behind model predictions in light of their own expertise, not only to safeguard against potential errors but also to foster trust and ensure actionability (Petch et al., 2022; Senoner et al., 2024; Abbas et al., 2025). More broadly, trust in AI-based clinical decision support depends on transparency, usability, clinical reliability, and familiarity with the system (Tun et al., 2025). Prior work further shows that clinicians actively negotiate AI recommendations against clinical judgment and patient-specific considerations (Sivaraman et al., 2023). To address these challenges, explainable AI (XAI) has garnered attention for its ability to enhance trustworthiness, informativeness, causality, confidence, interactivity, accessibility and fairness by providing insights into which factors drive model predictions (Ihongbe et al., 2024; Abbas et al., 2025; Gambetti et al., 2025). Furthermore, research suggests that explainability may indirectly support physicians’ intention to use AI by strengthening technology trust and perceived value (Liu et al., 2022). Among XAI approaches, post-hoc explanations are particularly relevant for complex black-box models because they do not require modifications to the underlying model and can therefore be applied while preserving predictive performance (Markus et al., 2021). Consequently, there is a growing demand for XAI in healthcare, as it supports both patient safety and professional accountability (Sendak et al., 2020) while also contributing to clinicians’ trust in AI-based decision support (Sivaraman et al., 2023; Tun et al., 2025). In addition to supporting understanding and trust, explainability can also contribute to the validation of AI-based clinical decision support in practice (Sivaraman et al., 2023). In high-stakes settings, clinicians need to identify potentially implausible predictions, detect misleading patterns, and assess whether model outputs align with clinical knowledge and the local care context (Petch et al., 2022; Sivaraman et al., 2023). Explanations can therefore support a form of local plausibility checking by helping clinicians compare model reasoning with their own expertise, recognize potential errors or biases, and decide when additional scrutiny or human override is necessary (Petch et al., 2022; Sivaraman et al., 2023; Tun et al., 2025). Beyond clinical considerations, the demand for XAI in healthcare is reinforced by both ethical and legal requirements. The European Commission’s Ethics Guidelines for Trustworthy AI emphasize principles such as human agency, transparency, and explicability, highlighting that AI systems must be understandable and interpretable to their users and those affected by their outputs (European Commission, Directorate-General for Communications Networks, C., and Technology, 2019). As a complementary regulatory framework, the EU AI Act establishes binding obligations for AI systems. Systems considered to be high-risk, which frequently encompass clinical applications due to their potential implications for patient safety, are subject to more stringent transparency, interpretability, and human oversight requirements (European Union, 2024). These measures are implemented to ensure that users can correctly interpret outputs and intervene when necessary (European Union, 2024).

To address this growing demand for transparency, there has been an increased adoption of common explanation methods in healthcare. However, only limited efforts have been made to involve clinicians in the method selection process or to conduct a systematic human-centered evaluation of the generated explanations, ultimately resulting in limited practical effectiveness (Sendak et al., 2020; Ihongbe et al., 2024; Gambetti et al., 2025). A review of 213 cardiac studies indicates that almost half of the studies did not evaluate explanations (Salih et al., 2024). The majority of the remainder employed a literature-grounded evaluation approach and a small portion applied quantitative metrics (Salih et al., 2024). Only 11% relied on feedback from healthcare professionals, often due to the time and cost required for clinician involvement in the evaluation process (Salih et al., 2024). Thus, there remains a notable lack of research investigating how healthcare professionals perceive different types of explanation, especially for tabular data (Bergomi et al., 2025).

To ground the investigation in a clinically relevant context, the present study examines XAI within a prototype of an early-warning system designed to detect imminent heart failure decompensation based on laboratory tabular data. Cardiovascular diseases remain the leading global cause of death, with heart failure affecting more than 64 million people worldwide and representing the primary cause of hospitalization among the elderly (Savarese et al., 2023; World Health Organization, 2025). Heart failure is a multifaceted condition, not only associated with significant morbidity but also with high mortality, reduced quality of life, and substantial healthcare costs (Savarese et al., 2023). Telemonitoring has the potential to alleviate the burden of chronic diseases such as heart failure on healthcare systems by enabling continuous patient monitoring and timely intervention in case of deterioration (Poncette et al., 2020). The combination of high-stakes decisions, complex patient data, and the need for actionable predictions provides a clinically relevant context for exploring how cardiovascular healthcare professionals perceive explanation methods and their capacity to support the understanding of, and trust in AI-assisted decision-making.

Building on this context, the present study seeks to address the following research question:

*RQ: How do cardiovascular healthcare professionals perceive and compare local post-hoc explanation methods regarding understanding, trust, and actionability in a prototype early-warning scenario based on tabular data?*

To examine how clinicians perceive and assess different explanations, this study employs an exploratory, qualitative, and human-centered evaluation of three methods: SHapley Additive Explanations (SHAP), Counterfactual Explanations (CFEs), and Anchors. For each method, an explanation was generated for the same model prediction based on a standardized patient case derived from real tabular clinical data. The evaluation was situated in a clinically grounded prototype scenario reflecting a future AI-supported early-warning system for the telemedical care of patients with chronic heart failure, while the present study focused on static explanation stimuli based on tabular laboratory data. Semi-structured interviews were conducted with 15 German cardiovascular healthcare professionals to compare and evaluate the explanations using the Explanation Satisfaction Scale (ESS; Hoffman et al., 2018). This enabled an in-depth exploration of cardiovascular healthcare professionals’ perspectives, experiences, and reasoning processes.

## 2 Related work

### 2.1 Importance of XAI in healthcare

The role of XAI in fostering trust and acceptance of AI in healthcare has been the subject of extensive discussion. Trust, however, is not only a technical property but also a relational and epistemic one, contingent on those involved and the knowledge being produced by the technology. In a qualitative study on computational phenotyping for rare disease diagnosis, Hallowell et al. (2022) demonstrated that stakeholders perceive trust in medical AI as twofold: firstly, as interpersonal trust between patients, clinicians, and developers; and secondly, as epistemic trust in the technology’s reliability and accuracy. The findings of the study demonstrate that transparent systems have the capacity to strengthen both dimensions, thereby supporting clinicians’ willingness to adopt AI tools in practice (Hallowell et al., 2022). Beyond trust, XAI has also been demonstrated to enhance clinical performance and decision quality. For instance, Sivaraman et al. (2023) examined interpretable treatment recommendations for sepsis management under different explanation conditions. Clinicians rated the AI as more useful and confidence-enhancing when feature explanations were provided alongside the AI recommendation, as opposed to the absence of such explanations (Sivaraman et al., 2023). In think-aloud sessions, participants placed a high value on explanatory evidence, with comparisons of alternative treatments being regarded as particularly persuasive for clinical decision-making (Sivaraman et al., 2023). Notably, the clinicians did not readily adopt or dismiss recommendations but rather engaged in a process of “negotiation,” weighing and prioritizing aspects of the AI’s suggestions to reach balanced decisions that integrated both AI insights and clinical judgment (Sivaraman et al., 2023). Similarly, Senoner et al. (2024) found that radiologists supported by explanations in the form of heatmaps achieved higher diagnostic accuracy compared to those using a black-box system. The incorporation of explanations aided clinicians in verifying predictions and enhanced overall task performance (Senoner et al., 2024). However, participants with access to explanations also overruled incorrect model outputs less frequently, suggesting that increased transparency may also promote overreliance (Senoner et al., 2024).

Despite the favorable impacts of explanations in AI-based medical systems, their integration into healthcare workflows remains inadequate. In a survey across 40 computing and healthcare professionals, Bernal and Mazo (2022) reported that only 27.5% of participants were provided with explanations pertaining to the decision-making processes and recommendations of routinely used AI systems. This lack of transparency and interpretability contrasts sharply with users’ need for understandable, actionable, and regulatory-compliant explanations. User-centered research in various healthcare disciplines has highlighted these concerns. For instance, Müller et al. (2021) found that explainability, the ability to oversee AI decisions, and maintaining professional accountability were key factors influencing the adoption of AI in dental diagnostics. Similarly, Poncette et al. (2020) emphasize interoperability, usability, regular staff training, and transparency as essential requirements for the use of AI in intensive care settings.

The findings of these studies indicate a persistent misalignment between the common design process of AI systems and the needs of end users, highlighting the importance of involving clinicians in the development and evaluation of AI systems. Poncette et al. (2020) found that more than half of the surveyed healthcare professionals expressed a desire to participate in the design of new digital technologies, reflecting the value they place on having a say in the tools they ultimately use. In alignment with this, Bernal and Mazo (2022) draw attention to communication gaps between researchers and end users, which render much AI research inaccessible and misaligned with clinical needs. The necessity of engaging clinicians, patients, and other relevant stakeholders throughout the research and development process is argued to be essential for ensuring that AI systems meet real-world clinical needs and consider the perspectives of diverse stakeholder groups (Bernal and Mazo, 2022).

### 2.2 Commonly applied explanation methods

In practice, certain explanation methods are used repeatedly across healthcare research, reflecting their popularity rather than systematic comparison or rationale. SHAP, Local Interpretable Model-agnostic Explanations (LIME), and Gradient-weighted Class Activation Mapping (Grad-CAM) emerge as the most frequently applied techniques (Salih et al., 2024; Abbas et al., 2025). SHAP and LIME are versatile across various data types, with SHAP particularly common for tabular data, while Grad-CAM is primarily employed in medical imaging (Salih et al., 2024; Abbas et al., 2025). Other explanation methods, such as attention mechanisms for sequential data, CFEs for actionable recommendations, and gradient-based methods like Integrated Gradients, are used less frequently but offer an alternative means of highlighting relevant features (Abbas et al., 2025). These trends illustrate that, despite the variety of available techniques, studies tend to rely on a relatively small set of established methods, forming a practical foundation for investigating their suitability and effectiveness in clinical contexts.

Ultimately, the actual value of XAI depends on how well it conforms to clinicians’ needs and cognitive processes (Gambetti et al., 2025). Preliminary research indicates that AI explanations frequently fail to align with clinicians’ perceptions of usefulness and are incongruent with clinical workflows, creating socio-technical gaps between explanations and user expectations (Ihongbe et al., 2024; Gambetti et al., 2025). The practical value of explanations depends on the clinical context, the expertise of the user, and the type of decision being made, thereby underscoring the necessity to tailor the presentation, detail, and timing of explanations to the end user (Abbas et al., 2025). Since there is no universally applicable explanation method that adequately caters to all contexts, user-centered evaluations are essential to explore clinicians’ preferences and identify which forms of explanation most effectively support their decision-making processes (Bergomi et al., 2025). However, the majority of research has thus far evaluated explanations primarily in isolation, without examining their interaction with end users (Longo et al., 2024). This oversight may hinder the effective adoption of AI-based decision support in healthcare (Gambetti et al., 2025).

### 2.3 Evaluation of AI explanations with healthcare professionals

Evaluation of XAI can be classified as functionally-grounded, which relies on computational proxies; human-grounded, which may take the form of simplified experiments with laymen; or application-grounded, which involves domain experts performing real tasks (Doshi-Velez and Kim, 2017). Existing evaluation studies are often constrained by the recruitment of lay participants or by overly simplified experiments that are not representative of real-world tasks (Senoner et al., 2024). In many cases, these limitations stem from the considerable resources required for application-grounded evaluations with domain experts (Gambetti et al., 2025). However, due to their fidelity to clinical workflows, application-grounded evaluations are particularly well-suited for medical XAI, safeguarding both clinical reliability and patient safety (Senoner et al., 2024; Gambetti et al., 2025). As the measure of explanation quality should be domain-specific, subjective, and qualitative, evaluation methods such as semi-structured interviews provide a flexible means to gather detailed insights from users about their experiences with the XAI system (Gambetti et al., 2025).

When conducting a human-centered XAI evaluation, studies often concentrate on a single method, neglecting to benchmark it against alternatives, thereby restricting insights into the most effective explanations for end users (Longo et al., 2024). A notable exception is the study by Ihongbe et al. (2024), who examined explanation methods in the context of AI-based diagnostic support for chest radiography. The survey-based study compared visual explanations generated by Grad-CAM and LIME for the two diagnostic scenarios of pneumonia and COVID-19. Clinicians evaluated the explanations in terms of clinical relevance, comprehensibility, and confidence in the explanations. Both methods were rated similarly in terms of clinical relevance and comprehensibility, although Grad-CAM was generally preferred for its coherency, perceived accuracy, and richer visual detail, which increased clinicians’ trust. While the study provides valuable comparative insights, it is constrained by its limited sample size and composition, with 26 clinicians participating, of whom only eight were radiologists. Further comparative insights were provided by Bergomi et al. (2025), who studied clinicians’ assessments of explainable-by-design versus post-hoc explanation methods for predicting COVID-19 hospitalizations from tabular clinical data. In a questionnaire-based experiment, clinicians reviewed ten real patient cases, each accompanied by model predictions and explanations generated with Bayesian Networks, SHAP, and AraucanaXAI. Participants indicated their level of agreement with the model’s prediction and rated the explanations based on two adapted items from the ESS regarding understandability and actionability. The findings revealed a positive correlation between understandability and actionability, with SHAP emerging as the favored method due to its perceived simplicity. While the study contributes to the understanding of clinician attitudes toward a selection of explanation methods, its limited sample size of ten participants restricts the generalizability of the results. Additionally, the restriction to only two ESS items may constrain the scope of the evaluation and fail to capture other salient aspects of explanation quality. Complementing these survey-based studies, Röber et al. (2025) explored clinicians’ perceptions of XAI within a broader qualitative investigation into their thoughts, expectations, and reservations regarding the use of AI in healthcare. Eleven semi-structured interviews were conducted with clinicians from various fields. While the majority of the interview questions addressed experiences with and attitudes towards AI, one section focused on the evaluation of explanations using a simplified, hypothetical dataset in an intensive care setting with dummy variables for predicting a binary outcome. The participants discussed three explanation methods: SHAP, CFEs, and weighted rules. SHAP was favored for its ease of interpretation, CFEs for offering deeper insight despite higher cognitive demand, and weighted rules for their resemblance to clinical reasoning, though less suitable for complex scenarios. As the evaluation of the explanations constituted only a minor element of the broader interview and relied on a hypothetical example without standardized evaluation measures, the findings remain exploratory. Collectively, these studies highlight the value of human-centered evaluations, while emphasizing the necessity for further research to explore diverse explanation methods in realistic clinical contexts and with broader, more representative clinician perspectives.

## 3 Methodology

In order to address the research question, we initially aimed for an application-grounded evaluation. However, as the prototype had not yet been deployed and the setting did not fully resemble performing a real task, this study is best described as a prototype-based, human-centered qualitative evaluation with domain experts and application-oriented elements. The semi-structured interview guideline was grounded in the ESS (Hoffman et al., 2018) and data were analyzed via qualitative content analysis (Mayring and Fenzl, 2019). Each participant evaluated three local explanations: SHAP, CFEs, and Anchors, each for the same real but standardized patient case with a model-predicted decompensation risk based on seven clinical parameters. We employed semi-structured expert interviews because this method enables the collection of open-ended data, allowing us to explore participants’ thoughts, feelings, and beliefs, and to probe personal issues that fixed questionnaires cannot capture. In healthcare research, semi-structured interviews are the most commonly used qualitative interview type and a well-established approach (DeJonckheere and Vaughn, 2019).

### 3.1 Prototype design

The web-based prototype employed in this study is an AI-supported early-warning system intended for the telemedical care of patients with chronic heart failure (see Section 1 of the Supplementary material). In the intended final system, cardiologists initially complete an onboarding process comprising an interactive in-app tutorial, a printed guide providing background information, and a self-assessment intended to support reflection and ensure thorough understanding of the system. Subsequently, they may register in the online portal and recommend the system to suitable patients under their care. Following the installation of the associated mobile application, patients are guided through their onboarding process in the form of an in-app tutorial. Thereafter, they can register in the app and submit a connection request to their cardiologist, who is instructed to accept only requests from their own patients. Upon acceptance of the request, the patient’s data become accessible to the cardiologist via the online portal.

Analogous to blood glucose meters, a novel, minimally invasive smart patch continuously measures multiple parameters in the patients’ blood. Once the patient has connected the patch to the mobile application via Bluetooth, any newly measured data are automatically transmitted to a central server where the AI model is deployed. The AI model predicts the probability of a patient’s condition deteriorating, using imminent decompensation as an example target, and expresses the risk as a percentage. As soon as new health data are available, the model recalculates the individual decompensation risk score, which is directly updated in the online portal. Routine checks are recommended to cardiologists in cases where the predicted decompensation risk is low (0–33%) and closer monitoring is advised for moderate decompensation risk (34%–66%). By default, risk scores above 67% trigger an alert to the attending cardiologist; this threshold being adjustable to minimize the potential for alarm fatigue.

It is important to note that the AI does not make any automatic decisions. Rather, it is conceptualized as a physician-in-the-loop decision-support tool. In instances where the AI model indicates a high probability of decompensation, the attending cardiologist is responsible for examining the data, interpreting the prediction in the context of the patient’s clinical status and medical history, and, if deemed necessary, contacting them. Consequently, the predicted risk score may inform high-stakes clinical decisions, including whether to intensify monitoring, request additional diagnostic information, adjust medication, schedule an earlier consultation, or advise urgent clinical assessment or hospitalization.

Since the development of smart patch technology was still in its infancy at the time of conducting this study, continuous monitoring data were not yet available. Therefore, the model was trained on individual laboratory test results obtained from routine examinations. The study cohort comprised 118 patients treated at a university hospital in Germany. The dataset encompasses seven parameters that the smart patch will be able to measure in the future: NT-proBNP, hemoglobin, lactate, potassium, creatinine, pH, and sodium. Prior to the collection and use of this dataset, ethical approval was obtained from the Ethics Commission Westphalia-Lippe on June 24, 2023 (reference number: 2023-141-f-S). A random forest classifier was trained on the dataset to generate the stimuli for this study. The defined target outcome was positive decompensation status, as indicated by medical record entries and confirmed by the administration of furosemide medication. Thus, the prediction problem was formulated as a binary classification task, with the resulting decompensation risk being derived from the predicted probability of the positive class. Subsequent to data cleaning, the final modeling dataset comprised 173 well-balanced observations (95 decompensated, 78 non-decompensated). To prevent data leakage, a strict patient-level split (70% training, 15% validation, 15% test) was implemented, and model stability was evaluated using 5-fold stratified cross-validation on the training split. On the independent test set, the final model achieved a technical performance of 90% accuracy, 84% precision, 91% recall, 88% F1-score, and 93% area under the receiver operating characteristic curve, establishing its technical plausibility for stimulus generation. These figures reflect proof-of-concept technical performance only. Given the limited sample size, substantial missing data, absence of longitudinal time-series information, and lack of external validation, the model is not adequate for real-world clinical deployment. Detailed reporting on preprocessing, alternative candidate algorithms, and hyperparameter tuning is provided in Section 2 of the Supplementary material.

### 3.2 Stimulus design

To explain the decisions of the random forest model, we selected three complementary explanation methods: SHAP, CFEs, and Anchors (Lundberg and Lee, 2017; Wachter et al., 2017; Ribeiro et al., 2018). Detailed information regarding the configuration of the explanation generation parameters is available in Section 3 of the Supplementary material. Each method represents a different explanation paradigm: attribution-based, example-based, and model-based, respectively (Markus et al., 2021). Attribution-based methods quantify the contribution of individual input features to a model’s prediction (Markus et al., 2021). In the case of Lundberg and Lee’s (2017) SHAP, feature importance values are assigned to individual predictions, offering insight into how each input influenced the model’s output. Example-based approaches elucidate model behavior by identifying or generating representative instances that illustrate the relationship between input variations and model responses (Markus et al., 2021). Specifically, CFEs by Wachter et al. (2017) identify minimal changes to input features that would alter the model prediction, offering actionable “what-if” scenarios. Model-based methods describe the reasoning of the underlying model by either leveraging the model itself or by developing a simplified surrogate that approximates its decision logic (Markus et al., 2021). Anchors, introduced by Ribeiro et al. (2018), provide rule-based explanations that specify which feature conditions must be met for the model to reach a particular prediction, highlighting the key factors driving the decision.

**Figure 1:**
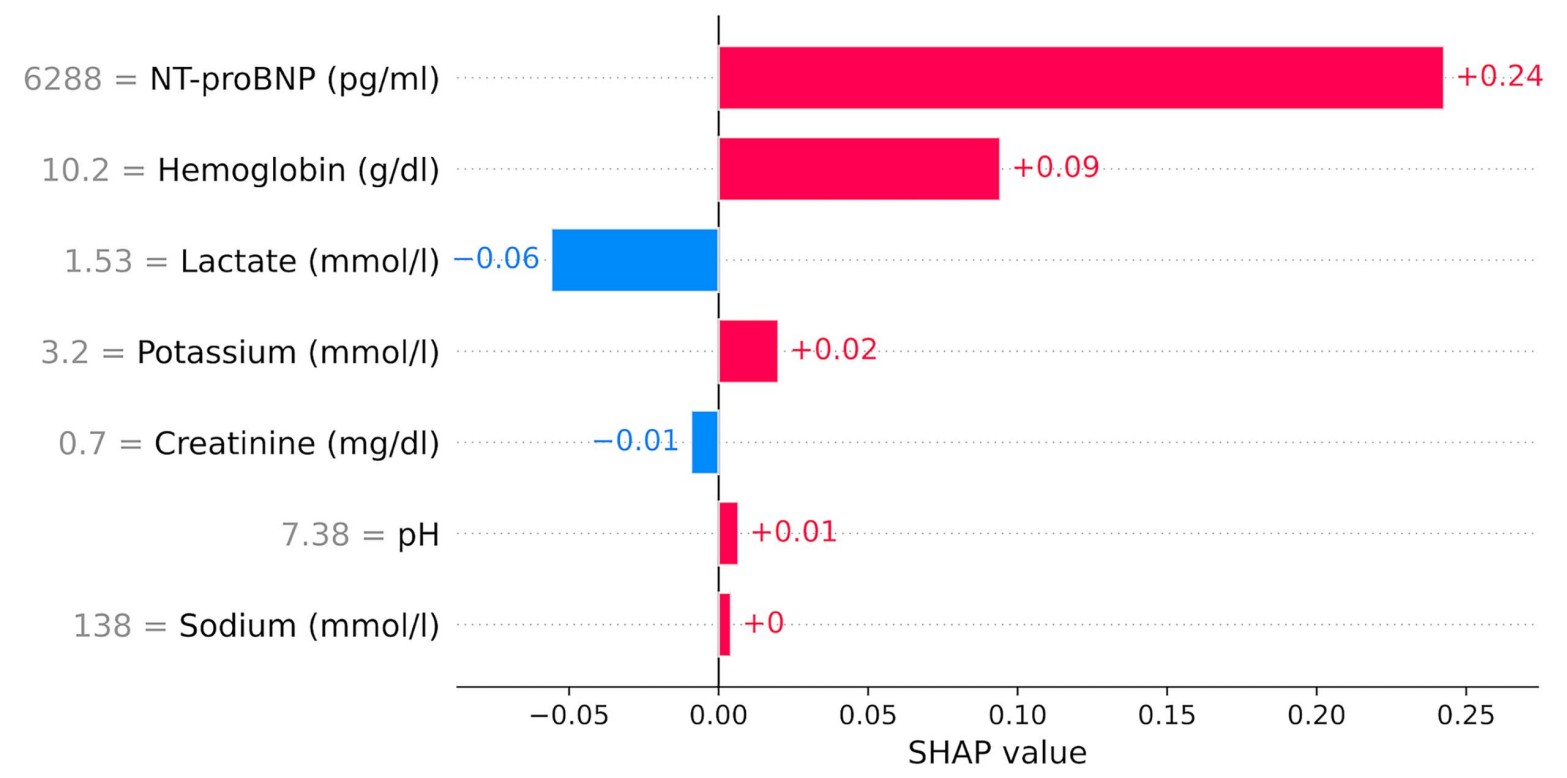
SHAP explanation used during the evaluation with cardiovascular healthcare professionals.

**Figure 2:**
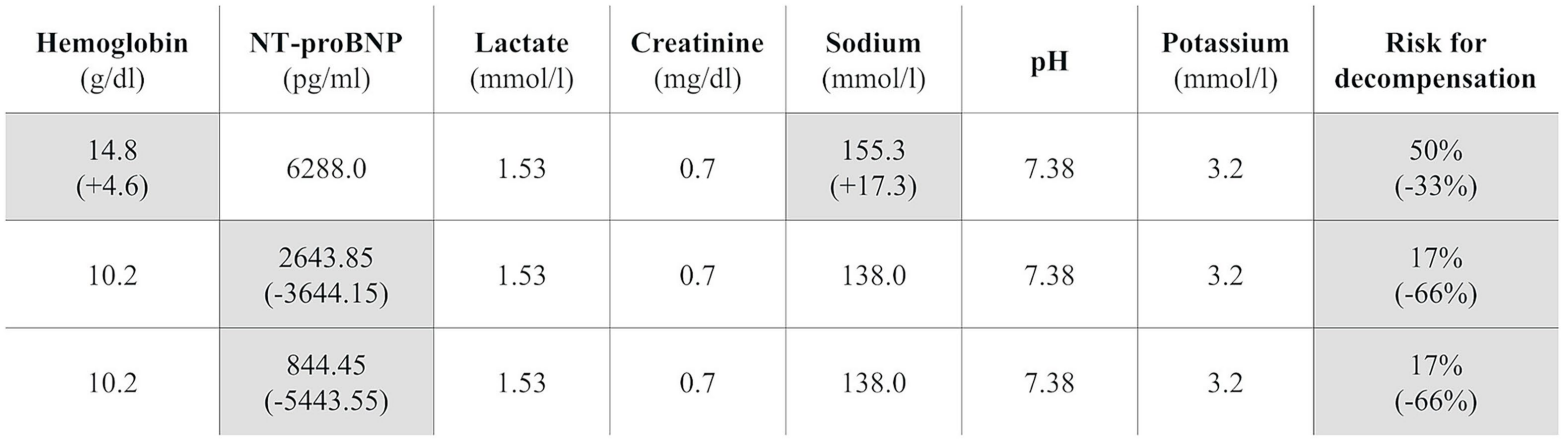
CFEs used during the evaluation with cardiovascular healthcare professionals as model-generated hypothetical “what-if” scenarios, though not as clinically validated treatment recommendations.

**Figure 3:**
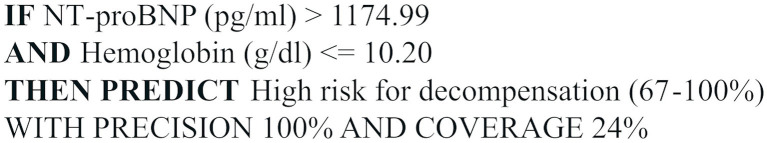
Anchors explanation used during the evaluation with cardiovascular healthcare professionals.

These explanation methods were chosen because they are model-agnostic, allowing flexible application to any type of model without requiring access to its internal structure (Markus et al., 2021; Abbas et al., 2025). Additionally, as they are applied post-hoc, these methods preserve the predictive performance of the underlying model by enabling the use of sophisticated models, which is particularly important in clinical settings where inaccurate predictions directly impact patients’ lives (Markus et al., 2021). All three explanation methods produce local explanations, enabling comprehensibility at the individual case level, which is essential for clinical decision-making and optimized patient care (Metta et al., 2024). Furthermore, the explanation methods employ a variety of visualization formats: SHAP provides diagrams (Figure 1), CFEs deliver numeric tables (Figure 2), and Anchors offer rule-based textual explanations (Figure 3). The model served to generate realistic explanation stimuli for the qualitative evaluation and was not intended to represent a clinically validated decision-support system. Prior to the interviews, the selected patient case and the corresponding explanation stimuli were reviewed by two physicians involved in the project to assess their clinical plausibility and face validity. To ensure a rigorous and unbiased clinical review, neither physician was involved in the development process of the AI model and prototype. The reviewers included: (1) a cardiologist with 42 years of professional experience in internal medicine and cardiology, who serves as the head of the interdisciplinary heart failure section at the German university hospital that provided the study dataset, and (2) a cardiologist practicing in a doctor’s office with 44 years of professional experience in internal medicine and cardiology. This expert evaluation ensured that the stimuli were adequate for discussion with cardiovascular healthcare professionals, while the study did not evaluate the clinical performance or safety of the underlying prediction model. These use-case specific explanations were then presented to cardiovascular healthcare professionals during the qualitative evaluation, ensuring diverse options for assessing different aspects of explanation quality. To the best of our knowledge, these techniques have not yet undergone a systematic, human-centered evaluation with intended end users in a clinically grounded scenario.

In healthcare, explanations are considered effective and supportive of trust in XAI systems when they are relevant, intuitively understandable, actionable, and aligned with the user’s expertise (Metta et al., 2024; Bergomi et al., 2025). Hoffman et al. (2018) formalized these criteria into the ESS, enabling a systematic, human-centered evaluation of explanations that captures the multifaceted requirements of cardiovascular healthcare professionals in clinical decision-making contexts. In this study, the ESS was applied within a prototype-based evaluation: the system should be understood as an AI-supported early-warning tool that is currently under development, not as an operational system. Participants therefore evaluated a clinically grounded scenario with corresponding explanation stimuli that simulated how such information could be presented in a future clinician-facing interface. Accordingly, the study focused on cardiovascular healthcare professionals’ perceptions of different explanation formats in a prospective use context.

### 3.3 Sample and interview structure

We conducted semi-structured expert interviews following best-practice guidance for qualitative interviewing in healthcare (DeJonckheere and Vaughn, 2019). After a brief introduction to the purpose and functionality of the developed prototype, the participants were posed a series of demographic questions regarding their professional background and experience with AI. This was followed by the presentation of an illustrative patient case that served as the basis for evaluating the three explanations (see Section 4 of the Supplementary material). The stimuli were displayed within the prototype, simulating how such explanations could appear in the clinicians’ future daily workflow. In the interviews, the three explanation stimuli were presented in varying orders across participants to reduce potential sequence effects. Because the final number of interviewees was not known at the beginning of data collection, a fully balanced presentation order could not be predetermined. We therefore used a pragmatic rotating sequence to vary the order of explanation stimuli across interviews. The first interview followed the order SHAP, CFEs, Anchors. In subsequent interviews, the starting method was rotated so that each explanation method appeared in the first position across the interview series. Residual order, learning, or fatigue effects can therefore not be fully ruled out. Similar to prior qualitative XAI studies with clinicians, participants received a brief introduction of each explanation before evaluating it. For instance, Röber et al. (2025) provided clinicians with an information sheet before the interview. In our study, the guidance was limited to the practical interpretation of the displayed explanation and did not constitute any in-depth technical training. We consider this consistent with a realistic scenario, as prior work emphasizes the need for onboarding and training when introducing clinicians to AI-supported decision tools and their explanations (Cai et al., 2019). Accordingly, the study assessed participants’ perceptions after brief guided orientation, rather than fully unaided interpretability. Subsequently, a set of questions guided by the ESS (Hoffman et al., 2018) were posed, before proceeding to the next stimulus. Thereby, the ESS was operationalized into seven dimensions, paired with open questions and further optional follow-ups (see Table 1). Finally, the interviewees were asked to provide an explicit preference ranking of the three explanations from 1 (favorite method) to 3 (least favorite method). The complete interview guide is available in Section 5 of the Supplementary material.

**Table 1:** Questions from the operationalized ESS, posed for each explanation.

Dimension	Question	Follow-up questions (optional)
Comprehension	To what extent does the explanation improve your understanding of the model prediction?	Which aspects of the explanation are particularly helpful or hindering to your understanding? Can you describe in more detail what the explanation clarified for you about the prediction and what remained unclear?
Sufficiency	How would you rate the level of detail in the explanation? Which aspects were explained well, and where would you have liked more detailed information?	Is there any information that you find too technical, brief, or general? Would you say that the explanation is too simplified or too complex, considering your information needs? Are some aspects of the explanation too superficial or overloaded for you?
Completeness	What additional information would you have expected or needed in the explanation in order to gain a complete picture of the AI model’s decision-making process?	In your opinion, are there any gaps or unanswered questions? How would you envision a “complete” explanation?
Actionability	Imagine using our online portal as part of your daily work routine. To what extent does the explanation convey how you can use the model prediction in patient care?	Can you imagine how you would use this explanation in a conversation with colleagues or patients? Would the explanation help you justify decisions better? Does the explanation provide any guidance on how to act in this situation?
Usefulness	To what extent is the explanation useful to your goals as a physician or to your decision-making regarding patient care?	To what extent does the explanation help you make, justify, or challenge medical decisions? In which situations would the explanation be particularly useful, and in which situations would it not be useful?
Accuracy	What information about the reliability or accuracy of the AI prediction do you obtain from the explanation?	Could you infer any limitations or uncertainties of the AI model from this explanation?
Trust	To what extent does the explanation help you recognize the situations in which you can trust the model predictions and those in which you should be wary?	Does anything about the explanation make you feel uncertain, or does anything help you gain trust in the predictions? What additional information would you need to build trust in a targeted manner?

We recruited and interviewed twelve practicing cardiologists, two heart failure nurses, and one internist with specialization in hematology and oncology. The inclusion criteria comprised routine interpretation of the seven laboratory parameters and regular involvement in decompensation risk management. Participants were recruited via professional networks and referrals. Accordingly, the study relied on a convenience sample, which may introduce selection bias and overrepresent participants with a particular interest in digital health or AI-supported clinical decision support. At the same time, eight participants reported no prior experience with AI in their professional work, suggesting that the sample was not limited to healthcare professionals already familiar with AI-based systems. Demographics and roles are summarized in Table 2. The interviews were conducted in German by two researchers with backgrounds in information systems and experience in qualitative interview methods. One interviewer was involved in the development of the prototype system, while neither interviewer had a clinical role or a direct relationship with the participants. The sessions lasted 27–43 min, were audio-recorded, and transcribed via Microsoft Teams. The data were analyzed using structured qualitative content analysis in two steps (Mayring and Fenzl, 2019). Coding was performed on the original German transcripts, and selected quotations were translated into English by the authors, partly supported by DeepL, for reporting. First, we employed deductive category application to the ESS dimensions for each explanation type and mapped positive and negative opinions. Second, we conducted an inductive refinement to capture emerging topics outside the ESS. We first double-coded eight of the 15 interviews independently using MAXQDA 2020. Intercoder reliability was satisfactory (Krippendorff’s *α* = 0.815). Discrepancies were discussed in a reconciliation meeting to reach consensus and finalize a shared codebook. The remaining seven interviews were divided between the two researchers and coded using the agreed codebook. The codebook and exemplary excerpts are available in Section 6 and Section 7 of the Supplementary material. Building on Guest et al. (2006), who report that around twelve interviews can already capture the vast majority of themes, we assessed our data for saturation and found that no substantively new codes emerged after the 13th interview. Given the study scope and the specialized target group of cardiology professionals, conducting 15 interviews provides an appropriate and sufficient qualitative sample.

**Table 2:** Overview of participants’ demographic and professional background.

Interviewee identifier	Role	Age	Professional experience in years	Experience with AI at work
C1	Cardiologist	49	25	None
C2	Cardiologist	32	4	None
C3	Cardiologist	64	36	None
C4	Cardiologist	68	40	Yes
C5	Cardiologist	54	25	Yes
C6	Cardiologist	44	16	Yes
C7	Cardiologist	69	40	None
C8	Cardiologist	27	1	Yes
C9	Cardiologist	69	43	Yes
C10	Cardiologist	55	30	None
C11	Cardiologist	38	11	Yes
C12	Cardiologist	61	30	None
H13	Heart failure nurse	48	15	None
H14	Heart failure nurse	51	5	None
I15	Internist	57	29	Yes

## 4 Results

In the following sections, we explore patterns that emerged from the interviews, organized into method-specific subsections. Each subsection reports representative quotations and results linked to the respective deductive ESS dimensions and inductive codes identified for SHAP, CFEs, and Anchors. The distribution of codes is shown in Table 3. To increase transparency, we report both the number of coded mentions and the number of participants contributing at least one coded segment to the respective code. In the text, these values are indicated in parentheses as coded mentions n | participant level n. Coded mentions refer to the total number of coded segments and may therefore exceed the number of participants, as one participant could contribute multiple segments to the same code. Participant-level counts indicate how many participants expressed a respective positive, negative or inductive perception at least once. In a few cases, participant-level counts do not sum to 15 because some participants did not express a clear position on a given dimension or provided ambivalent statements that were not assigned as a strong positive or negative assessment.

**Table 3:** Coded-segment and participant-level distribution of ESS items and inductive codes across explanation methods.

Item	SHAP		CFEs		Anchors	
	# positive	# negative	# positive	# negative	# positive	# negative
Comprehension	18|14	1|1	16|12	2|2	12|10	5|4
Sufficiency	13|10	7|5	11|11	5|4	10|10	6|5
Completeness	12|10	8|5	10|8	7|5	5|5	11|10
Actionability	16|12	4|3	9|9	6|5	11|10	4|3
Usefulness	13|10	4|4	12|10	9|5	11|11	2|2
Accuracy	12|9	5|5	9|8	7|7	4|4	8|8
Trust	12|11	4|4	13|12	3|3	8|7	6|6
Agreement	13|10	—	11|8	—	4|4	—
Value unclear	—	11|7	—	8|6	—	4|4
Representation	17|10	—	7|4	—	—	4|4
Overview	12|8	—	—	—	6|6	—
Time efficiency	8|5	—	—	11|7	4|4	—
Relevance	—	—	—	—	—	13|10
Therapy Recommendation	5|4	—	9|6	—	—	—
Patient consultation	4|4	2|2	2|2	2|2	3|2	—
Colleague consultation	3|3	3|2	2|2	1|1	2|1	—
Didactic purpose	—	—	5|4	—	1|1	—

In addition to the predefined ESS dimensions, several inductive codes emerged from the interview data, reflecting more applied and context-specific perceptions of the explanations. *Agreement* captures whether the explanation is consistent with participants’ own clinical reasoning or assessment of the example case. *Value Unclear* refers to instances of misunderstanding, such as confusion about the meaning of SHAP values, the interpretation of CFEs as sequential rather than independent scenarios, or uncertainty about the origin of Anchor thresholds. *Representation* describes the visualization format of each explanation (e.g., diagram for SHAP, table for CFEs, textual rule for Anchors), while *Overview* indicates whether the explanation provides a broad understanding at a glance. *Time Efficiency* addresses the speed and ease of comprehension, and *Relevance* refers to the perceived clinical significance of the information, as opposed to it being considered self-evident. *Therapy Recommendation* captures whether the explanation supports the derivation of specific treatment steps. *Patient Consultation* and *Colleague Consultation* refer to the perceived usefulness of the explanation in communication with patients and peers, respectively. *Didactic Purpose* describes the potential for educational use, particularly for familiarization with the AI model or for training less experienced healthcare professionals.

Across the ESS dimensions, SHAP was perceived most favorably overall, showing the highest number of positive mentions in nearly all categories, particularly *Comprehension* and *Actionability*. CFEs were evaluated as the second-best option, with relatively high ratings in terms of *Comprehension* and *Trust*, but with more critical feedback concerning *Usefulness*. Anchors received the least favorable assessment, evidenced by lower positive mention counts and higher negative feedback, particularly with regard to *Completeness*. Among the inductive codes, SHAP was also perceived most positively, especially in terms of *Representation* and *Overview*. CFEs received the most positive mentions for *Therapy Recommendation* and *Didactic Purpose*, while Anchors emerged as the only method to accumulate negative comments regarding *Relevance*.

The explicit preference ranking expressed by the 15 cardiovascular healthcare professionals aligns with the overall pattern observed in the interviews. SHAP was the clear front-runner, achieving the best mean rank (M = 1.26) and being placed first twelve times. CFEs followed with a mean rank of 2.20, while Anchors ranked last overall with a mean rank of 2.53 and were assigned last place in ten instances, indicating a marked preference gap between SHAP and the other methods.

### 4.1 SHAP

Participants consistently reported strong support for SHAP to understand the prediction of the AI model (18|14 positive mentions), and many felt that the explanations aligned with their clinical experience (13|10 mentions). One clinician explained, “From my day-to-day practice, I would also expect NT-proBNP to be most relevant. That matches what I would have guessed at first glance” (C8).

Several participants initially found the axis labels and units unclear, though this confusion was resolved with a brief explanation. “I have to admit, I don’t know what a SHAP value is. An explanation would help” (C1). Once clarified, the representation was described as intuitive by most.

The graphical layout, including the bars and color coding, was highlighted as a major strength (17|10 mentions). It enabled a quick overview (12|8 mentions) and time-efficient reading (8|5 mentions). One clinician summarized: “It considers many factors and presents them in a comprehensive yet clear way. The colors and bars give me a fast, well-rounded picture” (C2).

Perceptions of completeness (12|10 positive, 8|5 negative) and level of detail (13|10 positive, 7|5 negative) were mixed. While the included parameters were generally considered relevant and sufficient, participants recommended adding reference lines for normal or threshold values: “It would be complete for me if normal ranges were included” (H14) and providing a more dynamic view of time series data: “For several parameters, the trajectory matters more than a single value. For example, a steep rise” (C4). C7 also emphasized parameter value changes as more actionable than absolute risk probabilities: “The delta from the starting value tells me who is at risk. An 83% probability alone tells me little. If I know it has increased tenfold, I know to call the patient immediately or adjust treatment”.

Regarding the actionability with colleagues and patients (16|12 positive, 4|3 negative) and for clinicians’ own goals (13|10 positive, 4|4 negative), the sentiment was largely favorable. Some participants could readily imagine using the graphic to communicate prognoses or diagnoses to patients and colleagues and to inform therapeutic decisions. “I’d factor this into medication dosing and investigate issues like anemia or iron deficiency. These insights can guide concrete follow-up actions” (C4).

Finally, participants indicated that SHAP could help explain model accuracy (12|9 positive, 5|5 negative) and bolster trust in risk predictions (12|11 positive, 4|4 negative): “This graphic is essential to understanding how the probability is derived and how each factor contributes, which is very important for trust” (C2). Overall, SHAP’s bar-based visualization aligns with familiar clinical displays and enables the rapid identification of influential parameters: “In everyday practice, visualization beats text. With a bar chart, you immediately know what matters and can grasp it much faster than by scanning numbers” (C7).

### 4.2 CFEs

Participants generally found the CFEs helpful for understanding the prediction (16|12 positive, 2|2 negative), and many reported an alignment with their own clinical experience (11|8 mentions). One clinician noted, “This matches what we see in daily practice, NT-proBNP is the key driver of decompensation risk, so it makes sense that if it decreases, the risk would go down” (C8).

At the same time, several respondents (*n* = 8|6) initially misread the three example rows as a time series. They emphasized the need for an explicit explanation of the display logic. Another concern was the information density. The large number of digits led to perceived complexity and a higher time burden (*n* = 11|7). As one respondent put it, “In practice, physicians need to decide quickly and serve many patients, but this presentation doesn’t help with that” (C7).

Nevertheless, some appreciated the “what-if” tabular format for enabling more granular insights into how specific changes in parameter values affect predicted risk across scenarios, even if it takes more time to understand (7 mentions). One interviewee remarked, “It’s academically interesting to see that lowering NT-proBNP reduces risk less than increasing hemoglobin. Graphically, that’s well shown” (C5).

Views on the level of detail (11|11 positive, 5|4 negative) and completeness (10|8 positive, 7|5 negative) were cautiously positive. Several participants suggested offering more than three scenarios to present a fuller picture and clarify the influence of additional variables. One participant said, “From the second and third columns, I can tell NT-proBNP carries a lot of weight, but I can’t infer how much creatinine or lactate matters for the model’s decision” (C8).

Opinions were divided regarding actionability with patients and colleagues (9|9 positive, 6|5 negative) and usefulness (12|10 positive, 9|5 negative). Some (*n* = 5|4) saw strong didactic potential: “Absolutely for colleagues, and possibly educational for patients. If we adjust one parameter, you can see how another outcome changes. That’s great” (C6). C7 also suggested using CFEs in onboarding or tutorial phases for the whole system, where a small set of worked scenarios helps clinicians internalize how to read and apply predictions. Others, however, judged it too complex for patient conversations. “Patients respond better to curves or clear visuals. They don’t need numbers like 2,600 versus 3,600. They want to know if things will get worse, better, or stay the same” (C1).

For clinical decision-making, some participants perceived CFEs as potentially useful for structured what-if reasoning (*n* = 9|6): “I can see that if a given parameter changes, risk is reduced. This shows which therapeutic paths we should pursue to lower the patient’s risk” (C12). At the same time, several clinicians cautioned that CFEs could be misleading or clinically infeasible. For example, suggesting improvements in markers that are not directly actionable (“NT-proBNP doesn’t help you. You can’t influence it directly”, I15) or implying implausible changes (“A sodium of 155 would be incompatible with life”, C5). Others warned against naive therapeutic inferences (“This might prompt someone to give a transfusion to raise hemoglobin, which wouldn’t be the right step”, C5) and noted practical limits (“You can’t just raise hemoglobin ad hoc […] these are theoretical models”, H14). Perceived accuracy was mixed (9|8 positive, 7|7 negative), but trust in the predictions tended to improve (13|12 positive, 3|3 negative). Overall, participants described CFEs as useful supplementary and detailed lens within their potential future workflow.

### 4.3 Anchors

Participants generally reported improved comprehension with Anchors (12|10 positive, 5|4 negative), and some noted alignment with their professional experience (4|4 mentions). However, four clinicians criticized the wide risk interval (67–100%), arguing that finer granularity is needed, especially for high-risk decisions. One cardiologist stated, “With only two parameters and such a broad 67-100 band, this doesn’t guide me on who to prioritize first versus second” (C6).

A recurring concern was under-complexity. Many participants (*n* = 13|10) judged a single rule with two parameters as overly simplistic and offering little added value: “This doesn’t help me. I already knew that. I don’t need the AI to restate it” (I15). Another clinician added, “The patient is reduced to two lab values. They’re important, but it’s a very narrow view. I’d want to understand the algorithm and how it reaches this conclusion” (C1). Therefore, perceptions of detail and completeness leaned critical. Ten respondents had positive perceptions of detail, while six had negative perceptions. Five respondents had positive perceptions of completeness, while eleven had negative perceptions. One respondent said, “The system pulled out the two main parameters, but experienced cardiologists prefer seeing the broader set of data” (C3).

At the same time, respondents appreciated the very fast overview (*n* = 6|6) and time efficiency (*n* = 4|4): “It’s simple, just three lines and it’s immediately clear, even without explanation” (C10). Opinions were more favorable for actionability and practical use (actionability: 11|10 positive, 4|3 negative; usefulness: 11|11 positive, 2|2 negative), with some noting suitability for non-specialists and brief communications: “If it’s a non-specialist area I don’t know well, this shows me what the AI is looking at and which parameters matter. That’s good” (C7); “For example, I could imagine this working well in a discharge letter or for a general practitioner. Short and succinct” (C8). Clinicians also valued the quick threshold cues that prompt closer attention: “It gives me cut-offs I can keep at hand to know when to look more carefully” (C10) and “We need pragmatic tools, and two parameters can be a pragmatic start” (C6).

However, perceived accuracy (4|4 positive, 8|8 negative) and trust (8|7 positive, 6|6 negative) were mixed to negative, largely due to the perceived simplicity. “No, this leaves out a lot of what I actually want to know” (I15). Overall, while Anchors offer speed and pragmatic thresholds, most interviewees felt that a two-parameter rule lacked sufficient depth and did not meaningfully extend their existing clinical knowledge.

### 4.4 Combined methods

Eight interviewees advocated combining multiple types of explanations, ideally all three or at least pairing SHAP with CFEs. Most agreed that SHAP is excellent for an initial, quick overview, and is often sufficient. However, CFEs should be available for cases where predictions seem uncertain because they allow for a more in-depth, scenario-based analysis. As one clinician put it, “If it isn’t immediately convincing, I would definitely add [CFEs] and even [Anchors] on top” (C6). Another interviewee suggested a layered workflow: “Have [SHAP] pop up by default and allow me to click into [CFEs] when I need more detail” (C4). In the broader context, one participant summarized: “[Anchors] can provide initial cues for interpreting the risk score. [SHAP] is most helpful for the current snapshot. [CFEs] support dynamic “what-if” reasoning. Clinicians may lean toward [CFEs] for complex decisions, whereas [SHAP] may suffice for routine follow-ups” (C11). Finally, five interviewees emphasized that building trust in such systems is a long process that depends on continuous use. Appropriate explanations can facilitate, but not replace, this gradual development of trust.

## 5 Discussion

In response to our research question, the findings suggest a layered configuration rather than a single favored explanation method. SHAP was most preferred in this exploratory evaluation and may therefore be suitable as the default option. Its familiar bar charts provide a quick graphical overview that cardiovascular healthcare professionals can easily understand under time pressure, and this is often sufficient for routine follow-ups. When predictions are questionable or high-stakes, CFEs provide the necessary depth for “what-if” reasoning. While many participants found them conceptually valuable, they were also described as being too complex and time-consuming for typical use. They are better suited for on-demand drilldowns and are promising as a training or information tool for colleagues and potentially patients. Furthermore, CFEs may inform structured clinical reasoning about potential follow-up actions, but their clinical use requires careful interpretation and feasibility constraints to avoid misleading inferences. Anchors were regarded as clear and pragmatic, offering straightforward thresholds that elicited attention. However, they were also deemed to be overly narrow, since a two-parameter rule risks oversimplification or a false sense of understanding. Overall, the participants advocated for the provision of all three explanations as options: SHAP by default, with one-click access to CFEs and, where appropriate, Anchors, so clinicians can match the depth of explanation to clinical need.

The overarching pattern that emerges from the interviews suggests that cardiovascular healthcare professionals value flexibility in choosing the amount and form of explanation they receive. The findings indicate a delicate balance between providing sufficient detail and avoiding information overload. Despite CFEs being perceived as time-consuming, most clinicians valued their addition to SHAP, especially in critical situations where extra insights can support confident decision-making. Individual preferences were also found to be a contributing factor. Although Anchors were ranked lowest overall, they were selected as the first preference by two participants, indicating that opinions regarding explainability also vary among clinicians from the same fields. These results align with prior studies showing clinicians’ preference for simpler explanations and the need for adjustable explanation depth that combines quick overviews with the option for detailed insights (Abbas et al., 2025; Röber et al., 2025). Consistent with our findings, the clinicians participating in the interview study conducted by Röber et al. (2025) favored SHAP for its clarity, regarded CFEs as insightful but demanding, and indicated that the preferred method is contingent on the clinical context. This observation suggests a certain degree of transferability, given the inclusion of clinicians from diverse medical fields in their study. Röber et al. (2025) further observed that, irrespective of the explanation method, clinicians tend to prefer visual explanations for their immediacy and accessibility, as they can reduce cognitive load and potential language barriers. Overall, the consistently favorable assessments of SHAP across all evaluation dimensions in our interviews indicate that it is not only a technically versatile method capable of handling diverse data types but also one that is genuinely valued by healthcare professionals. This pattern aligns with its widespread adoption in the medical XAI literature (Salih et al., 2024; Abbas et al., 2025), where SHAP is frequently discussed as a versatile method for explaining model predictions across different data types and clinical contexts. However, it is important to note that this does not imply that SHAP will necessarily outperform other methods in every evaluation. Different medical specialties may have distinct requirements, and other explanation methods may be more appropriate depending on the type of data or decision task. For instance, in the domain of medical imaging, gradient-based techniques such as Grad-CAM are tailored specifically to visual data and have demonstrated strong clinician acceptance (Ihongbe et al., 2024). Thus, while SHAP’s broad applicability and high acceptance make it a practical baseline for XAI in healthcare, optimal method choice should always be guided by the clinical context and end user feedback.

In our interviews, the consensus among all participants was that the provision of explanations enhanced the value of the AI-based decision support tool. While explainability is a contributing factor to trust, it is not the sole element that determines its establishment. The participants’ statements suggest that trust in AI is an experience-based process that develops gradually through consistent system performance, targeted onboarding, and exposure to concrete clinical cases. The necessity of developing familiarity with the system over time to cultivate confidence in its outputs was emphasized, thereby echoing the notion that trust must be learned rather than granted. This observation is consistent with the findings of prior studies by Winter and Carusi (2022), Hallowell et al. (2022), and Röber et al. (2025), who characterized trust in medical AI as a multifaceted and iterative process. Winter and Carusi (2022) argue that commonly acknowledged components of trust, such as algorithmic transparency and the ability to justify model outputs, are necessary but insufficient on their own. Instead, they assert that trust in AI is established through collaboration and negotiation between clinicians and developers during implementation and validation. Similarly, Hallowell et al. (2022) found that clinicians’ initial lack of trust in AI-based diagnostic tools stems from the novelty of the technology and limited hands-on experience. Their interview participants described trust as being established through repeated use and validation, with clinicians learning trust through experience and technology itself earning trust as its reliability is empirically demonstrated. Röber et al. (2025) likewise observed that clinicians tend to build trust through certified use and repeated exposure, while distinguishing it from responsibility, as ultimate accountability for medical decisions remains with the clinician. Consequently, fostering trust in AI-supported clinical decision-making also requires iterative engagement, user training, and continuous validation within real-world practice. In this regard, end user training emerges as a foundational component, as also emphasized by Röber et al. (2025). Current XAI is often engineered for technical audiences (Liao and Varshney, 2022), but clinical deployment requires plain-language legends, reference ranges, concise tutorials, and a growing library of patient cases to normalize usage. The provision of explanations for the explanations, which may take the form of targeted training sessions or embedded guidance, is essential to ensure correct interpretation of explanations and to prevent misconceptions. For instance, a prevalent misunderstanding in our interviews was the assumption that CFEs represent temporal sequences instead of distinct scenarios, indicating that clearer annotation or prior explanation is needed. At the same time, only few participants inquired in detail about the meaning of SHAP values, suggesting that extensive technical instruction is not always necessary and may even increase cognitive burden. This suggests that cardiovascular healthcare professionals require a practical rather than a profoundly technical understanding of the underlying methods. Accordingly, the design of training and explanatory materials should aim to provide sufficient conceptual grounding to ensure correct interpretation, while avoiding information overload and maintaining efficiency within clinical workflows.

Building on this, participants further emphasized that, in addition to the conventional goals of XAI, such as trust and accuracy, the practical utility in daily workflows is of equal importance. According to this perspective, explanations are considered valuable only if they integrate seamlessly into the cognitive and temporal workflow of the recipient, rather than existing as detached add-ons. A well-designed, layered XAI interface can help cardiovascular healthcare professionals manage larger patient panels without sacrificing decision quality by streamlining routine assessments and reserving depth for edge cases. To achieve such integration, it is essential that explanations align not only with model logic but also with clinical reasoning and feasibility. For instance, it was noted by some participants that certain counterfactual suggestions, such as increasing hemoglobin levels, may not be immediately actionable and could imply inappropriate interventions if misinterpreted. In that sense, the critical feedback on CFEs reveals a safety-relevant design requirement. This perspective is consistent with the findings of Röber et al. (2025), who similarly stressed the need for explanations to be seamlessly integrated into existing clinical workflows and for close collaboration between developers and healthcare professionals to ensure practical adoption. This underscores the importance of context-aware, clinically meaningful explanations and human-centered evaluations with real end users, focusing on the practical application of XAI tools in future clinical workflows.

While the participants of our study primarily emphasized the potential benefits of XAI integration, the existing literature also points to possible unintended effects. Prior research has cautioned that seemingly persuasive explanations, even for incorrect model outputs, may lead to unwarranted trust or overreliance on AI systems (Bergomi et al., 2025). While our participants regarded Anchors as overly simplistic, Röber et al. (2025) similarly cautioned that SHAP can also create a false sense of understanding and reinforce confirmation bias. The authors thus advocate for explanations that encourage critical engagement rather than passive acceptance. This underscores the need for meticulous design, end user training, and human-centered evaluation when incorporating XAI into clinical practice.

However, a practical challenge in evaluating XAI configurations is the sequencing of real-world clinical testing and regulatory approval. Evaluation in live clinical practice generally requires the AI system to already be certified as a medical device (European Union, 2017). At the same time, obtaining said regulatory approval, governed by frameworks such as the European Medical Device Regulation and the EU AI Act, requires demonstrating system transparency and accountability in advance (European Union, 2017, 2024; Amann et al., 2020). This introduces an operational challenge, as gathering empirical evidence on how clinicians interpret these explanations ideally benefits from real-world deployment, which is restricted without prior regulatory clearance. Consequently, while prototype-based evaluations with the intended target group, such as the one conducted in this study, cannot guarantee regulatory approval, clinical safety, or human oversight adequacy, they serve as a valuable intermediary step. In early development lifecycles, these insights may inform transparency-oriented design before advancing to formal clinical and regulatory validation.

Recent research also suggests that the trade-off between predictive performance and interpretability may be less pronounced than commonly assumed. In particular, models that are interpretable by design, such as Generalized Additive Models (GAMs), have been shown to achieve competitive predictive performance while maintaining transparency (Kruschel et al., 2026). When feasible, such inherently interpretable approaches should therefore be preferred over post-hoc explanation methods, as they generally provide more reliable and faithful explanations of the underlying model behavior.

Based on these findings, our study offers several contributions for research and practice. Firstly, it provides a human-centered evaluation of three complementary explanations with cardiovascular healthcare professionals within a clinically grounded prototype scenario. Secondly, the study proposes practical heuristics for workflow-aware XAI interface design in early-warning systems based on tabular laboratory data. Based on the findings from the conducted interviews, we propose a configuration that prioritizes a rapid visual overview through SHAP, preserves access to actionable detail when questions arise (CFEs), and offers simple thresholds where they add clear value (Anchors). This layered approach enables efficient yet flexible interactions, could foster understanding and trust over time, and might empower cardiovascular healthcare professionals to act more confidently on AI predictions in an intended care context. Finally, such design considerations may inform transparency-oriented discussions for emerging regulatory requirements concerning transparency and accountability in medical AI systems.

Nonetheless, this study is not without limitations. The comparison was restricted to three post-hoc, model-agnostic, and local explanation methods. The selection of these methods was deliberate, aiming to represent a diverse range of explanation types, including an attribution-based, an example-based, and a model-based approach (Markus et al., 2021). The explanations encompass different forms of representation, including a graphical diagram (SHAP), a numerical table (CFEs), and a textual rule (Anchors). Furthermore, variations in the level of detail manifest in the number of parameters incorporated into the explanations and the overall complexity of the presented information. This design choice enabled a controlled comparison across distinct explanation styles within the same clinical context. However, other commonly applied explanation methods for tabular data in the healthcare domain, such as LIME (Salih et al., 2024; Abbas et al., 2025) or GAMs which are interpretable by design (Kruschel et al., 2026), may also be suitable but were not considered in this study. Future research should therefore extend the comparison to additional explanation methods in order to examine whether the observed preferences remain consistent across a broader range. In addition, participants were shown only one example explanation per method. Future studies should therefore include multiple examples, including edge cases, to improve representativeness and allow a more comprehensive evaluation of how explanations perform under varying conditions. The present study was conducted as a prototype-based evaluation with the intended target group. However, participants evaluated a standardized scenario and explanation stimuli rather than an operational tool embedded in routine clinical practice. As the system is still under development, the assessment simulated a possible future clinician-facing workflow and did not yet include continuous smart patch data transmission, real-time risk score updating, or adjustable alert thresholds. Future studies should therefore evaluate how the explanations are perceived within the complete end-to-end telemonitoring workflow based on validated model predictions and over repeated clinical use. Furthermore, participants were presented with static, tabular explanations rather than real-time telemonitoring data. Therefore, the findings may not fully capture responses to time series data. Future studies should evaluate explanations using time series or multimodal data. Additionally, subsequent studies could explore adaptive interfaces that tailor the amount and depth of explanation to the user’s expertise and the complexity of the clinical task. Moreover, the assessment relied primarily on qualitative evaluation through semi-structured interviews, focusing on outcomes such as perceived usefulness, satisfaction, and intention to use. While this approach enabled an in-depth understanding of participants’ perspectives, it limits representativeness and may not reflect broader trends across the healthcare community. Future research could adopt mixed-methods approaches that combine interviews with larger-scale surveys or experimental studies and complement these qualitative measures with quantitative interaction-based metrics, such as decision accuracy, time required, likelihood to deviate from model recommendations, or the ability to detect model errors (Zhou et al., 2021). Furthermore, experimental comparisons between different explanation setups (e.g., single XAI method, multiple XAI methods, or no explanation) could help determine whether combining explanations improves understanding or instead leads to cognitive overload, longer decision times, or reduced trust due to conflicting explanations. Longitudinal studies may further examine how clinicians’ trust in AI systems and the role of explanations in validating model performance evolves with repeated use over time. Finally, the sample comprised a convenience sample of 15 German cardiovascular healthcare professionals, which restricts the generalizability of the results across medical specialties and application contexts. Future work should therefore include clinicians from a wider range of specializations and healthcare settings to examine whether similar preferences emerge across different target groups and clinical contexts.

## 6 Conclusion

This study examined how cardiovascular healthcare professionals perceive and evaluate three different AI explanations in a prototype early-warning scenario for chronic heart failure care. The findings indicate that, in isolation, none of the presented explanations universally meet clinical needs. Although SHAP was favored most, participants expressed a preference for a layered configuration of SHAP, CFEs, and Anchors. SHAP may serve as an intuitive default for rapid interpretation, CFEs provide on-demand depth for intricate cases, and Anchors deliver concise cues when simplicity is required. However, it should be noted that employing CFEs to derive therapeutic steps requires the generation of scenarios under clinically meaningful constraints and careful interpretation to avoid inappropriate clinical interventions. Collectively, the three explanations may help cardiovascular healthcare professionals to balance efficiency, comprehensibility, and action ability. Beyond preferences for specific explanation methods, participants’ statements suggest that trust in AI develops gradually through familiarity, reliable performance, and appropriate onboarding. Training end users and providing explanatory support are therefore crucial to ensure correct interpretation while avoiding cognitive overload. A workflow-aware XAI design that integrates transparency into daily routines has the potential to enhance both usability and acceptance. The study offers empirically informed implications for the design of early-warning systems based on tabular laboratory data, emphasizing human-centered and context-sensitive explainability. A multifaceted approach may foster trust and acceptance while aligning with emerging regulatory requirements for transparency and accountability. Ultimately, future XAI designs should aim to support healthcare professionals in making confident, informed, and responsible decisions in their everyday medical practice.

## Data Availability

The datasets presented in this article are not readily available because of confidentiality reasons (regarding the modeling dataset and complete interview transcripts). The Supplementary material contains a prototype screenshot, a comprehensive description of the modeling pipeline, the explanation generation process, and the patient case alongside the complete interview guide, codebook, and translated excerpts.
