# Supplementary material for: Prescribing a dose of transparency: a qualitative evaluation of AI explanations with cardiovascular healthcare professionals
Source: Front Artif Intell. 2026 Jun 29;9:1830201. doi: 10.3389/frai.2026.1830201 (PMC13357571; doi:10.3389/frai.2026.1830201)
Supplement: Supplementary file 1 [file Data_Sheet_1.pdf]

frontiers

Supplementary Material

1 Prototype

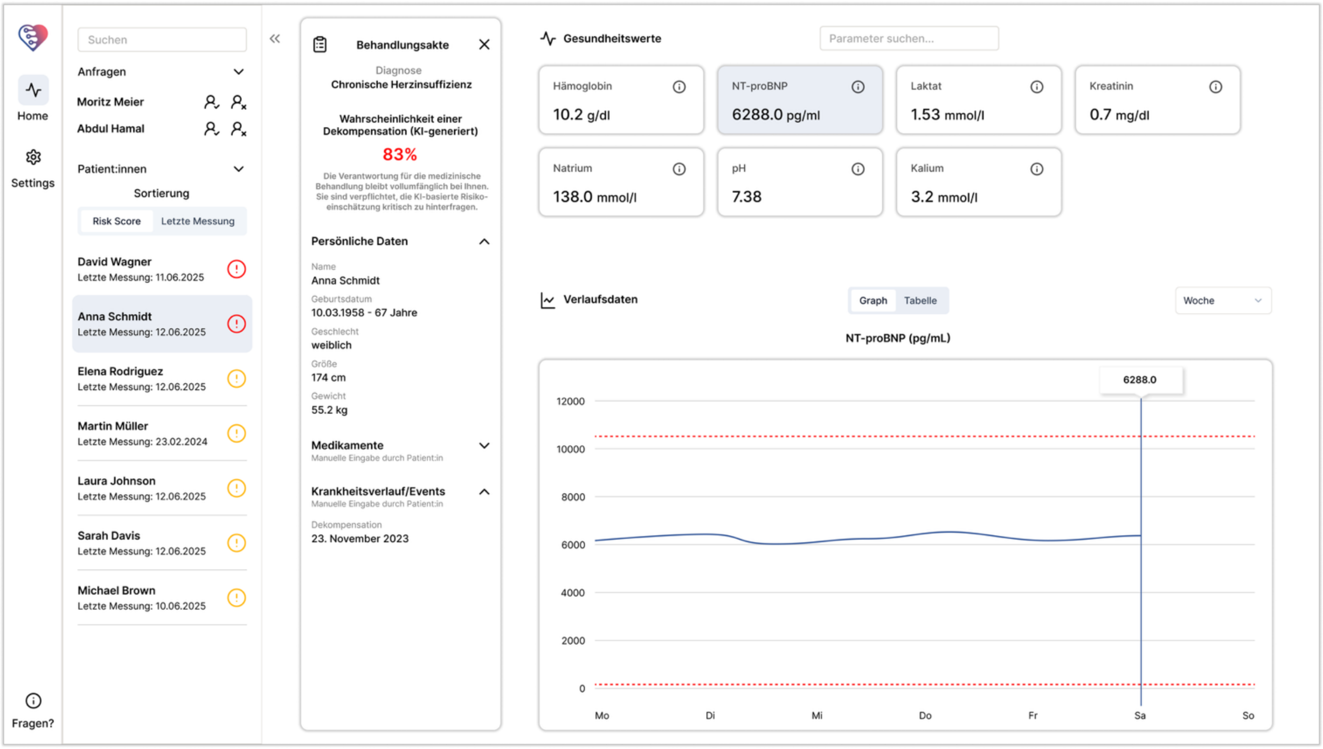

Supplementary Figure 1. The web-based prototype employed in this study is an AI-supported early-warning system intended for the telemedical care of patients with chronic diseases (explanations are shown in place of the trend graph when clicking on the AI-predicted decompensation risk score).

2 Modeling Pipeline

The dataset comprised routine blood test results from 118 patients, corresponding to 303 individual measurements, which included the seven laboratory parameters that the smart patch is intended to measure in the future: NT-proBNP, hemoglobin, lactate, potassium, creatinine, pH, and sodium. To prevent data leakage from repeated measurements, the data were partitioned using a strict patient-level split into 70% training, 15% validation, and 15% test sets. Of the 303 observations, 36 were excluded after performing outlier detection using interquartile range thresholds. As the dataset relied on opportunistic routine laboratory sampling prior to the initiation of continuous monitoring, data sparsity was high, and 94 observations needed to be excluded for containing more than 70% missing values. To address the remaining missing values, the data were scaled using the min-max method and subsequently, k-nearest-neighbor imputation was applied. Thereby, each method was fit on the training set, and the fitted transformation was subsequently applied to both the validation and test data to avoid information leakage. Given that the 173 remaining observations were well balanced, with 95 being classified as decompensated, no additional balancing technique was applied.

Several candidate classifiers were explored, including XGBoost, AdaBoost, random forest, decision tree, k-nearest neighbors, logistic regression, multilayer perceptron, naive Bayes, and support vector machine models. A random forest classifier was selected for the final proof-of-concept configuration because it provided the most suitable performance profile for generating explanation examples. For the random forest, hyperparameters were tuned using a random search with 50 trials. The search space covered maximum depths of 3-8, tree counts of 50-200 in increments of 50, and minimum samples per leaf of 3-7. The final configuration used a maximum depth of 8, 100 trees, and a minimum of 3 samples. Model stability during development was evaluated using 5-fold stratified cross-validation on the training split. The selected configuration yielded a proof-of-concept performance of 0.3 log-loss, 90% accuracy, 84% precision, 91% recall, 88% F1-score, and 93% area under the receiver operating characteristic curve on the test set. However, these values should not be interpreted as evidence of clinical prediction validity. Given the limited dataset, considerable missing data, absence of continuous time-series information, and lack of external validation, the model is not adequate for real-world telemonitoring or clinical deployment. Therefore, the fixed patient case and explanation stimuli generated from this model were reviewed by two project physicians for clinical plausibility and face validity before being used in the interview study.

3 Explanation Generation

Local post-hoc model explanations were generated for individual model predictions using three methods: SHapley Additive Explanations (SHAP), Counterfactual Explanations (CFEs), and Anchors. For SHAP, explanations were computed using the TreeExplainer from the shap package with an interventional feature perturbation strategy. The calculated Shapley values were visualized as a bar plot displaying all seven features. CFEs were generated using the dice_ml package. The "random" search method was deployed to generate three counterfactuals per sample, targeting the opposite outcome class. To ensure clinical plausibility, feature perturbation was strictly bounded by physiological ranges: 6-18 g/dl for hemoglobin; 30-100,000 pg/ml for NT-proBNP; 0.3-5.6 mmol/l for lactate; 0.5-7 mg/dl for creatinine; 128-170 mmol/l for sodium; 7.32-7.84 for pH; and 3.2-7 mmol/l for potassium. These thresholds were provided by a project physician to ensure representativeness. Anchors were computed using the AnchorTabularExplainer from the anchor package. Since the underlying model returned class probabilities for binary decompensation risk, the prediction probabilities were discretized into three risk categories prior to anchor generation: low (<33%), medium (33-66%), and high (>66%). Anchor explanations were constructed to meet a precision threshold of 98% and coverage of at least 5%.

4 Patient Case

Supplementary Table 1. Patient case data used for generating the stimuli.

Parameter	Value
Age	67 years
Sex	Female
Height	174 cm
Weight	55.2 kg
NT-proBNP	6288.0 pg/ml
Hemoglobin	10.2 g/dl

Lactate	1.53 mmol/l
Potassium	3.2 mmol/l
Creatinine	0.7 mg/dl
pH	7.38
Sodium	138.0 mmol/l

5 Interview Guide

[Expressions of gratitude for the participation and brief personal introductions of the interviewers.]

Description of the application scenario using the prototype image (Supplementary Figure 1)
The KardioInterakt system is an AI-supported early warning system designed to assist cardiologists in the telemedical care of patients with chronic heart failure. The system comprises an app for patients and an online portal for physicians. Employing a novel, minimally invasive smart patch, seven parameters will be continuously measured in the blood via a small abrasion (comparable to blood glucose meters for diabetics): hemoglobin, NT-proBNP, lactate, creatinine, sodium, pH, and potassium. We understand that this selection of parameters is quite minimalistic for a comprehensive assessment of health status. Subsequent iterations of the system will allow for the integration of additional sensors, thereby facilitating the incorporation of additional pertinent health data. However, this evaluation focuses only on the parameters currently measured by the smart patch. Leveraging time series data, AI will be employed to predict the probability of impending decompensation as a percentage value for each patient. For the sake of transparency, we must disclose that at this stage, continuous data collection with the smart patches has not yet been achieved. Consequently, the data used for model training consist of individual laboratory measurements from blood tests. Therefore, the planned temporal perspective cannot yet be taken into account at the current stage of development. Instead, the model makes its decisions based solely on snapshot data. Patients do not have access to the risk of decompensation predicted by the AI.
[Brief tour of the online portal for physicians.]
Please note that the AI model does not make any decisions autonomously. The responsibility for decision-making lies exclusively with the attending physician. In instances where there is a high probability of decompensation, the attending physician is obligated to review the available data and, if deemed necessary, contact the patient concerned. The AI model is a so-called "black box," meaning that it is not possible to trace how the AI's decisions are made. The only inherently available information is the percentage value predicted by the AI model. However, various explainability methods can be employed to decompose the influence of the parameters on the model's individual prediction for each patient. The proposed approach entails the incorporation of an explanation within the online portal, accessible by clicking on the AI prediction. In this interview, the objective is to assess which of the three explanations best conveys the AI-predicted probability of decompensation in a comprehensible manner, thereby fostering acceptance and trust. I will provide a concise overview of each explanation and subsequently pose a series of questions about them. Please note that the same questions will be reiterated for each of the explanations. Do you have any questions before we begin? If any questions arise during the interview, please feel free to address them at any time.
[Inquiries regarding the signing of the consent form and the agreement of the interview participant to the recording.]

Demographic characteristics of the interview participant
First, could you please introduce yourself briefly, including your age, profession, and professional experience, if you are willing to share that information?

Do you have experience with AI, particularly in your professional life?

Description of the medical case study using the prototype image (Supplementary Figure 1)
We would like to demonstrate all of the explanations using the medical case study that I emailed you in advance. The case study concerns a female patient with chronic heart failure. Information on her age, height, and weight can be found in the treatment file ("Behandlungsakte"). The respective values for the seven parameters measured by the Smart Patch are shown in the top right ("Gesundheitswerte"). The AI predicts an 83% probability of decompensation.

Explanation 1: SHAP (Figure 1)
This explanation provides a visual representation of the influence that each of the seven parameters had on the probability of decompensation predicted by the AI. Each bar in the chart represents one parameter, with the corresponding values listed adjacent to it. The longer the bar, the greater the influence of this value on the model prediction. The parameters are listed in descending order according to their influence. Red bars indicate an increase in the predicted probability of decompensation (e.g., NT-proBNP). Blue bars indicate a decrease in the predicted probability of decompensation (e.g., lactate). The SHAP values are additive, and the sum of all SHAP values corresponds to the difference between the individual prediction (83%) and the average model prediction (i.e., the average probability of decompensation across all patients in the training dataset). Do you have any questions regarding this explanation?
[Questions from Table 1.]

Explanation 2: CFEs (Figure 2)
This table shows the health data from the case study listed in three rows underneath each other. Only the values highlighted in blue changed, and the difference from the original value is shown in brackets. For instance, hemoglobin increased by 4.6, from 10.2 to 14.8. The three rows are independent of each other, i.e., they do not represent a chronological progression but rather different hypothetical "what if" scenarios. The last column shows the impact that changes in the values would have on the predicted probability of decompensation. In the first scenario, for instance, an increase in hemoglobin and sodium would reduce the predicted probability of decompensation by 33%, from 83% to 50%. The second scenario shows that a reduction in the NT-proBNP value would result in a reduction in the predicted probability of decompensation to 17%. The third scenario shows that an even greater reduction in NT-proBNP does not result in any further improvement in the predicted probability of decompensation. In other words, this explanation shows which changes in values would result in a lower predicted probability of decompensation. Do you have any questions regarding this explanation?
[Questions from Table 1.]

Explanation 3: Anchors (Figure 3)
This explanation is presented as an if-then rule. It shows which two values are particularly decisive: NT-proBNP and hemoglobin, in this case. If the conditions of the rule are met, i.e. if the NT-proBNP value is above 1174.99 and the hemoglobin value is 10.2 or lower, the AI model will predict a high probability of decompensation. This equates to a percentage value between 67 and 100 percent. Other parameters, such as pH or potassium, can influence the exact percentage within this range (e.g., from

70% to 80%), but they cannot push it below 67%. The rule has a precision of 100%, meaning the model consistently predicts high risk when these conditions are met, and coverage of 24%, meaning it applies to about a quarter of similar cases in the data. Note that this rule was created to explain this specific model prediction. In other cases, different rules with other key factors may apply. For instance, if the predicted probability of decompensation were low for a patient, the explanation might instead involve unremarkable NT-proBNP and stable hemoglobin values or completely different parameters. Do you have any questions regarding this explanation?
[Questions from Table 1.]

Ranking
Finally, you can see the three explanations side by side. Could you please rank them in order of preference and explain your reasoning behind the decision?

Is there anything else you would like to discuss that hasn't been covered in the questions so far?

[Thanks and farewell.]

6 Codebook

Supplementary Table 2. Codebook for the qualitative content analysis.

Explanation	Code	Explanation	Anchor examples
Anchors	Comprehension (positive and negative)	Captures whether the explanation helped participants understand how the model arrived at the prediction and what the key drivers were. Negative codes reflect confusion, misinterpretation, or missing interpretive cues (e.g., unclear labels)	**Positive:** "*That's helpful because it further differentiates which ones are based on which parameters, which the entire system is based on." C4* **Negative***: "This, what it has given me, is just not helpful to me, because I already knew that. So, the AI doesn't need to tell me that again." I15*
Anchors	Sufficiency (positive and negative)	Refers to the perceived depth and level of detail of the explanation for the given clinical task. Negative codes capture perceptions of being too shallow, too dense, or containing irrelevant/distracting information.	**Positive:** *"Well, I think that this story basically explains itself, especially when you look at the NTProBNP value." C3* **Negative:** *"As I said, I would be interested to know what further influence the other parameters have. And the prediction of decompensation, to what extent they change it further." C11*
Anchors	Completeness (positive and negative)	Describes whether participants felt the explanation provided all information needed to make sense of the prediction in context. Negative codes reflect missing	**Positive:** *"No, I think that would be sufficient, and I don't think it will be easy due to the time factor, I won't pursue it further, nor will I likely seek any more information." C2*

		elements participants expected (e.g., reference ranges, trends, additional variables).	**Negative:** *"…and secondly, it is too simplistic for me." I15*
Anchors	Actionability (positive and negative)	Captures whether the explanation suggested how the prediction could be used in practice, for example in communication with patients or colleagues or in follow-up steps. Negative codes reflect a lack of practical guidance or barriers to using the explanation in real workflows.	**Positive***: "There is a good point of reference, so it is a support in the decision-making process." C8*<br>**Negative:** *"Yes, that doesn't really help me move forward. Who should I look at first, and who should I look at second?" C6*
Anchors	Usefulness (positive and negative)	Refers to how well the explanation supported clinicians' goals (e.g., monitoring, triage, decision justification, learning). Negative codes capture limited relevance to clinical objectives or perceived low added value beyond existing expertise.	**Positive:** *"I think we always need a pragmatic approach, and that's very pragmatic here, so to speak. There are two parameters, and if I find them good at first glance, then I think you can work with them, yes." C6*<br>**Negative**: *"That's far too little for me. I already knew that, as general knowledge…" I15*
Anchors	Accuracy (positive and negative)	Captures whether and how the explanation informed participants about the reliability of the AI prediction (e.g., plausibility, uncertainty, consistency with clinical expectations). Negative codes reflect doubts about correctness, plausibility, or concerns about misleading/infeasible implications.	**Positive:** *"I think so, I know the values and can therefore rely on the fact that the values are unusual" C11*<br>**Negative**: *"The spectrum is relatively broad. That is very, very imprecise" C3*
Anchors	Trust (positive and negative)	Refers to whether the explanation helped participants judge when to rely on the AI output versus when to be cautious. Negative codes capture reduced confidence,	**Positive:** *"If it seemed completely strange to me, then I could decide whether I wanted to trust it." C8*<br>**Negative**: *"In the context of my clinical experience, if I were to say that I had no*

		perceived oversimplification, or a lack of cues needed to calibrate reliance.	*knowledge of the subject matter, I would find that very difficult." C1*
Anchors	Agreement	Captures whether participants perceived the explanation as consistent with their own clinical reasoning and assessment of the case. Negative instances reflect explicit mismatch with clinical expectations.	*"So I can compare it again with my clinical experience." C8*
Anchors	Value Unclear	Captures confusion about what the explanation means or how to interpret it (e.g., unclear SHAP values, CFEs misread as sequential scenarios, unclear Anchor threshold origins). It codes moments where interpretive support was missing or insufficient.	*"Not really satisfied, because you just can't put it together. This risk profile, where does the value of 1,174 come from? The AI calculated that, right?" H13*
Anchors	Representation	Refers to comments on the explanation's presentation format (e.g., SHAP bar chart, CFE table, Anchor rule text) and how this format affects interpretability. This includes preferences for visual vs. tabular vs. rule-based displays.	*"Yes, absolutely, although of course, if I only have those two parameters, then 67 to 100 is a wide range." C6*
Anchors	Overview	Captures whether the explanation provides a broad, at-a-glance understanding of the prediction and key drivers. It reflects perceived "quick orientation" rather than deep inspection.	*"Yes, it's like a mathematical equation. If this, then that; if it's to be reduced to two parameters, then that's explanation enough for me. Very good. Easy to grasp at a glance." C10*
Anchors	Time Efficiency (positive)	Refers to perceived speed and effort required to understand the explanation in a real clinical workflow. It captures statements about cognitive load, time pressure, and suitability for fast decision-making.	*"That's explained, it's only three lines, so it's easy to understand." C10*

Anchors	Relevance	Captures whether the information provided is perceived as clinically meaningful and decision-relevant versus trivial, self-evident, or distracting. It reflects added value beyond existing professional knowledge.	*"Well, that's how it is for me now, let's say as a cardiologist, only a little additional information. What I wouldn't have predicted, and I don't need an explanation either, because it corresponds to clinical experience. No surprise, let's put it that way." C5*
Anchors	Patient Consultation	Refers to perceived usefulness of the explanation for communicating risks, rationales, or next steps to patients in an understandable way. It includes suitability for shared decision-making or explanation of prognoses.	*"Could you explain to patients, for example, why you want to give them iron supplements, or why you want to or need to change their heart failure medication. I think it's also easier for patients to understand if you tell them that you know from certain models, or that based on the course of the disease, certain values will be exceeded, and I believe that would increase acceptance among patients and possibly also among doctors, as they would then also respond to these things." C1*
Anchors	Colleague Consultation	Refers to perceived usefulness of the explanation for professional communication with peers (e.g., handovers, referrals, documentation, case discussions). It includes use in conveying rationale succinctly to other clinicians.	*"Yes, I would say that something like that would be appropriate for a doctor's letter, for example, or for a general practitioner, I could well imagine something like that" C8*
Anchors	Didactic Purpose	Captures the explanation's perceived educational value, such as onboarding to the system, training less experienced clinicians, or building intuition about model behavior through examples. It includes use as a tutorial or learning aid beyond immediate decision support.	*"Yes, because I would know which parameters to use. These are things outside my field of expertise, that I know nothing about, but then I know what the AI looks at and which parameters are important. That's good, yes." C7*

CFEs	Comprehension (positive and negative)	Captures whether the explanation helped participants understand how the model arrived at the prediction and what the key drivers were. Negative codes reflect confusion, misinterpretation or missing interpretive cues (e.g., unclear labels)	**Positive:** *"It helps me to understand, to comprehend, to try to think like it, because, as the system wants to think, of course, the NTProBNP value is a very decisive value in assessing the question of whether there is decompensation or not." C3* **Negative**: *"I don't understand where the system ultimately draws the line, where it says OK, at an NTProBNP of 5000 or 6000 I have a high probability of decompensation, but now at only 2600 I only have 17%. I don't find that directly comprehensible." C2*
CFEs	Sufficiency (positive and negative)	Refers to the perceived depth and level of detail of the explanation for the given clinical task. Negative codes capture perceptions of being too shallow, too dense, or containing irrelevant/distracting information.	**Positive:** *"Completely sufficient, at least for us, because we are used to thinking in terms of heart failure. I'll leave it open for someone who is not from the field of cardiology to decide whether this is immediately understandable at first glance." C12* **Negative**: *"...I think it's too extensive." C8*
CFEs	Completeness (positive and negative)	Describes whether participants felt the explanation provided all information needed to make sense of the prediction in context. Negative codes reflect missing elements participants expected (e.g., reference ranges, trends, additional variables).	**Positive:** *"Yes, I don't know what's missing. So it's complete." C14* **Negative**: *"No, there are other parameters that are still deviating, so creatinine is fine. Of course, you could also consider potassium, which is very low, and that should actually be taken into account as well." C5*
CFEs	Actionability (positive and negative)	Captures whether the explanation suggested how the prediction could be used in practice, for example in communication with patients or colleagues or in follow-up steps. Negative codes reflect a lack of practical guidance or barriers to using the explanation in real workflows.	**Positive:** *"Yes, I already told you that I see the greatest strength of such a system in the fact that I can prioritize and that the system sorts the bad ones at the top and the good ones at the bottom." I15* **Negative**: *"As I said, this tabular presentation has no therapeutic benefit for me at this point." C5*

CFEs	Usefulness (positive and negative)	Refers to how well the explanation supported clinicians' goals (e.g., monitoring, triage, decision justification, learning). Negative codes capture limited relevance to clinical objectives or perceived low added value beyond existing expertise.	**Positive:** *"Yes, as I said, I think that's right, I think that's right, that degree in relation to NTProBNP. Since this decrease in the probability of decompensation is already there, you also have the figures in front of you, so that's impressive, yes." C1*<br>**Negative**: *"That's not how it works in practice, that's what I would say. Yes, it's just a mathematical game, but it's not how it works in practice." C10*
CFEs	Accuracy (positive and negative)	Captures whether and how the explanation informed participants about the reliability of the AI prediction (e.g., plausibility, uncertainty, consistency with clinical expectations). Negative codes reflect doubts about correctness, plausibility, or concerns about misleading/infeasible implications.	**Positive:** *"Yes, a little bit, to identify the pitfalls of AI." C7*<br>**Negative**: *"No, no, as I said, there aren't really any limits, because I have basically the same probability between 2608 and 1500. To be honest, I don't see any limits at all." C7*
CFEs	Trust (positive and negative)	Refers to whether the explanation helped participants judge when to rely on the AI output versus when to be cautious. Negative codes capture reduced confidence, perceived oversimplification, or a lack of cues needed to calibrate reliance.	**Positive:** *"Yes, yes, if something comes up, because I don't understand what it's doing right now, then either the model isn't good or the patient has something that wasn't in the training set." C8*<br>**Negative**: *"So, based on the explanation? No, not really." C10*
CFEs	Agreement	Captures whether participants perceived the explanation as consistent with their own clinical reasoning and assessment of the case. Negative instances reflect explicit mismatch with clinical expectations.	*"No, that's clear and understandable to me. From a medical point of view, the percentages will remain as they are." C2*

CFEs	Value Unclear	Captures confusion about what the explanation means or how to interpret it (e.g., unclear SHAP values, CFEs misread as sequential scenarios, unclear Anchor threshold origins). It codes moments where interpretive support was missing or insufficient.	*“Exactly, yes, that would be the point, that I thought I should now visualize a timeline.” C8*
CFEs	Representation	Refers to comments on the explanation’s presentation format (e.g., SHAP bar chart, CFE table, Anchor rule text) and how this format affects interpretability. This includes preferences for visual vs. tabular vs. rule-based displays.	*“But in order to compare the different assessments or percentages, it makes sense to use a table.” C2*
CFEs	Time Efficiency (negative)	Refers to perceived speed and effort required to understand the explanation in a real clinical workflow. It captures statements about cognitive load, time pressure, and suitability for fast decision-making.	*“You have to imagine the doctor in his practice, who wants to make quick decisions. He has to. He has to see a lot of patients and wants to do justice to everyone, and that’s why they don’t need this chart.” C7*
CFEs	Therapy Recommendation	Captures whether participants felt the explanation supports deriving or prioritizing concrete therapeutic actions or follow-up steps. It includes both direct treatment implications and actionable clinical next steps.	*“They would support me 100 percent, because if we have accurate records, then I have control, so to speak, over whether my therapeutic measures are effective or not.” C12*
CFEs	Patient Consultation	Refers to perceived usefulness of the explanation for communicating risks, rationales, or next steps to patients in an understandable way. It includes suitability for shared decision-making or explanation of prognoses.	*“Absolutely, absolutely once for colleagues, and I was just thinking, maybe also educational for patients, right?” C6*
CFEs	Colleague Consultation	Refers to perceived usefulness of the explanation for professional	*“I think that would be useful, just to check hemoglobin levels, perhaps talk to the family*

		communication with peers (e.g., handovers, referrals, documentation, case discussions). It includes use in conveying rationale succinctly to other clinicians.	*doctor again about iron supplementation, for example, something like that." H14*
CFEs	Didactic Purpose	Captures the explanation's perceived educational value, such as onboarding to the system, training less experienced clinicians, or building intuition about model behavior through examples. It includes use as a tutorial or learning aid beyond immediate decision support.	*"If that's good and the probability of decompensation decreases, then how important is good holistic therapy, not just heart therapy, but also, for example, giving iron to increase hemoglobin levels.*<br>*I think this can be clearly seen from this table and from the program, so it's also useful for us doctors in terms of teaching." C6*
SHAP	Comprehension (positive and negative)	Captures whether the explanation helped participants understand how the model arrived at the prediction and what the key drivers were. Negative codes reflect confusion, misinterpretation or missing interpretive cues (e.g., unclear labels)	**Positive:** *"That helps a lot in understanding this NTProBNP of 6288, which is very high, as one would think in the clinic. The patient is probably already decompensated. For that reason alone, based on this one value." C4*<br>**Negative**: *"That remains a bit cryptic too. She said that it's not clear how the algorithm behind it works, but ultimately, as I said, there's the question of what time period this refers to and so on, so there are still a few things left unanswered." C1*
SHAP	Sufficiency (positive and negative)	Refers to the perceived depth and level of detail of the explanation for the given clinical task. Negative codes capture perceptions of being too shallow, too dense, or containing irrelevant/distracting information.	**Positive:** *"It is clear, which is very important, and does not overload the reader with information." C2*<br>**Negative**: *"Yes, I agree, this is a selective analysis. But here, and this was also shown earlier in the example, this graphical representation of how the values are developing, we are on a downward trend, which means that the forecast is strongly influenced, or it is a diminishing problem due to a measure that has already been identified. And so this is just the current situation, which*

			is explained here, but not a dynamic view. That's what strikes me." C11
SHAP	Completeness (positive and negative)	Describes whether participants felt the explanation provided all information needed to make sense of the prediction in context. Negative codes reflect missing elements participants expected (e.g., reference ranges, trends, additional variables).	**Positive:** *"That's enough for me; the display is fine, showing the probability of decompensation. The weighting of the various parameters is shown. I don't need any more information." C5*<br>**Negative**: *"So for me, it would be complete if the reference values were included." H14*
SHAP	Actionability (positive and negative)	Captures whether the explanation suggested how the prediction could be used in practice, for example in communication with patients or colleagues or in follow-up steps. Negative codes reflect a lack of practical guidance or barriers to using the explanation in real workflows.	**Positive:** *"Yes, this is important for us as an assessment of an interim period. Assuming that the patient was hospitalized or treated as an outpatient, is now at home, and provides us with this data, I can say very precisely how the course of treatment is progressing, what the patient's values are, and whether they are developing in the right or wrong direction. And from this point of view, this is a complete and very good monitoring system. But the whole thing also always takes away a little from the progress monitoring." C12*<br>**Negative**: *"So at the moment, I would say that, with or without AI, NTProBNP is of course a very important value for me, so that I would say at the moment that, from a clinical point of view, it only benefits me to a limited extent. I don't really see the advantage of AI here yet, and it's somehow incomprehensible how AI arrives at this 83% figure." C1*
SHAP	Usefulness (positive and negative)	Refers to how well the explanation supported clinicians' goals (e.g., monitoring, triage, decision justification, learning). Negative codes capture limited relevance to clinical objectives or perceived low	**Positive:** *"Yes, it is extremely important because we monitor these patients' progress precisely according to the parameters listed here. And that's why it's great, also in connection with the graph shown at the beginning, where you show a curve that follows the course of time. That provides me*

		added value beyond existing expertise.	*with progress monitoring. So this snapshot in connection with progress monitoring is perfect." C12*<br>**Negative**: *"For me, that would be a snapshot. However, heart failure is a chronic condition, so I would feel that something was missing." H14*
SHAP	Accuracy (positive and negative)	Captures whether and how the explanation informed participants about the reliability of the AI prediction (e.g., plausibility, uncertainty, consistency with clinical expectations). Negative codes reflect doubts about correctness, plausibility, or concerns about misleading/infeasible implications.	**Positive:** *"Yes. Yes, what... If the NTPproBNP were very low and it still said 85%, I would say that doesn't fit." C9*<br>**Negative**: *"No, I can't, because NTProBNP is clearly so dominant here and the lactate value is so important for the most severe cases that the other factors are secondary, right? That's why I can't say how good the AI is and how good that is." C7*
SHAP	Trust (positive and negative)	Refers to whether the explanation helped participants judge when to rely on the AI output versus when to be cautious. Negative codes capture reduced confidence, perceived oversimplification, or a lack of cues needed to calibrate reliance.	**Positive:** *"I would say that the data you provide helps me to trust the system." C3*<br>**Negative**: *"After all, that seems to be the case, or at least that's what this 83% figure would suggest as a final value, but that's also what I know from my clinical experience, so to be honest, AI would be of little help to me here." C1*
SHAP	Agreement	Captures whether participants perceived the explanation as consistent with their own clinical reasoning and assessment of the case. Negative instances reflect explicit mismatch with clinical expectations.	*"So from my everyday professional life, I would also have chosen NTProBNP, which would have been very relevant for me. So it actually coincides with what I would have thought at first glance." C8*
SHAP	Value Unclear	Captures confusion about what the explanation means or how to interpret it (e.g., unclear SHAP values, CFEs misread as sequential	*"Well, to be honest, I don't know what SHAP value is. I honestly don't know what it is. Of course, that could have been explained, or an explanation would have been good. Because,*

		scenarios, unclear Anchor threshold origins). It codes moments where interpretive support was missing or insufficient.	*of course, I'm talking about these numbers, this 0.24, and I don't really know what kind of unit that is. Of course, compared to some other things, it's significantly more, but ultimately it remains unexplained what it actually is." C1*
SHAP	Representation	Refers to comments on the explanation's presentation format (e.g., SHAP bar chart, CFE table, Anchor rule text) and how this format affects interpretability. This includes preferences for visual vs. tabular vs. rule-based displays.	*"Overall, I find the entire system or structure to be very clear and the information well presented. As I said, the color coding certainly helps as well." C2*
SHAP	Overview	Captures whether the explanation provides a broad, at-a-glance understanding of the prediction and key drivers. It reflects perceived "quick orientation" rather than deep inspection.	*"I find it very, very clear and appealing, and it also allows you to quickly get your bearings, especially when you're looking through the parameters for several patients." C4*
SHAP	Time Efficiency (positive)	Refers to perceived speed and effort required to understand the explanation in a real clinical workflow. It captures statements about cognitive load, time pressure, and suitability for fast decision-making.	*"In everyday life, it's already the case that you tend to have too little time rather than too much, which also applies here when you're examining a patient. Ultimately, a certain simplicity and reduction of information makes perfect sense, and I think that's what this presentation offers." C2*
SHAP	Therapy Recommendation	Captures whether participants felt the explanation supports deriving or prioritizing concrete therapeutic actions or follow-up steps. It includes both direct treatment implications and actionable clinical next steps.	*"So, the risk of decompensation in dynamic observation helps me in treating this patient. Should medication be adjusted, or is it actually inevitable that the patient will have to be hospitalized and then compensated here? The goal is to prevent that. That's why I find it helpful. Then I can guide myself." C11*
SHAP	Patient Consultation	Refers to perceived usefulness of the explanation for communicating risks, rationales, or next steps to patients in an understandable way. It includes suitability for shared	*"Yes, and that's why I like it so much. So, I have a patient in front of me, or I called them, and I can show the first graph, for example. Here you can see the NTProBNP value, which has risen, and that's why there's a red bar*

		decision-making or explanation of prognoses.	*here, and you could also show that to patients who are competent and who want to know, right? We can say, look here, yes, you can see that it's conceivable" C7*<br><br>*"Yes, not with patients, because I think that would be even more confusing for them." C8*
SHAP	Colleague Consultation	Refers to perceived usefulness of the explanation for professional communication with peers (e.g., handovers, referrals, documentation, case discussions). It includes use in conveying rationale succinctly to other clinicians.	*"The idea is that it should make things easier for the attending physician or the study nurse, who then passes the message on to the physician. There is imminent danger. And that's how it should be" C9*<br><br>*"I wouldn't have to explain much to a colleague if I said that the NTProBNP is over 6000, which is obvious." C5*
Combined Methods		Captures explicit preferences for providing multiple explanation types and/or a layered interaction design (e.g., SHAP as default overview with optional drill-down to CFEs and/or Anchors). It reflects the perceived complementarity of methods to match explanation depth to clinical uncertainty and task context.	*"So I would like to see 1 and 2. Like, let's see what we can click on, or maybe the first one pops up immediately and then you can go to 2 for more information, or whatever." C4*

7 Translated Excerpts

7.1 SHAP

I: To what extent does this explanation help you better understand and follow the prediction of the AI model?

P: The NT-proBNP value is now a well-established value. For me, it is very plausible and often correlates very well with clinical probability. We have patients with structural heart disease, and in those patients, low hemoglobin can also be associated with reduced cardiac performance. That is also very plausible to me. So, this image seems plausible.

I: So, you can compare it with your own experience and would say that it matches?

P: Yes. These medical findings are, in part, very non-specific. You can have changes in lactate or potassium values in different diseases, and changes in hemoglobin in different diseases. In everyday life, we would always interpret these values in the context of the overall clinical picture of the patient. We examine the patient clinically, we ask questions, we perform technical examinations such as an echocardiogram or an X-ray, and then we add laboratory values to create plausibility for a disease. In that sense, this is a building block. These are values that we use in everyday life, and therefore it seems plausible to use them here as well. It is not something I would consider implausible. It is roughly how we think as well: NT-proBNP is an extremely good value, hemoglobin is very important, and lactate and creatinine also play important roles.

7.2 SHAP

I: To what extent does this graphical breakdown help you better understand and follow the prediction of the AI model?

P: I definitely find the color coding and the graphical representation useful and understandable. The SHAP value, so the 0.24, does not really tell me anything at first. But with this breakdown I can understand how strongly the model weighted the values. At a rough glance, it is understandable to me how the probability comes about based on these values.

I: Would you need the additional explanation of the SHAP value, or would you also manage with the graphical breakdown alone?

P: That is a good question. For understanding, I would find it important that this information is included somehow or maybe stored behind the value 0.24. From my own work in clinical research, I often see that representations and graphics are prepared in a way that makes them look nice. So, some decoding, or some understanding of the values, is important. At least if I were working with the system, I would want to inform myself more closely so that I can better understand and interpret the information.

I: Can you say a little more about how you would assess the depth or level of detail of this graphical explanation?

P: Overall, I find the whole system or the whole structure nicely arranged and the information well presented. The colors certainly help as well. In everyday practice, it is usually the case that you have too little time rather than too much. So, when you are looking through a patient, a certain simplicity and reduction of information is definitely useful. And I think this visualization provides that.

7.3 SHAP

I: Imagine using our online portal in your everyday work. To what extent does this explanation give you an idea of how you could use the AI model’s prediction in patient care?

P: Yes, that would be quite simple. I would know that I probably need to bring the patient in, because I do not understand why she has anemia. That is a practical problem, and it needs to be clarified. Maybe she is also simply overhydrated, then we need to act. But we cannot act blindly, because she already has hypotension, so at least loop diuretics would not simply be the answer. I would probably need to look more closely and consider starting other medications. But if I saw this, I would basically

know: she needs to be seen. It is not an emergency, given that lactate is not yet a major problem, but she should probably be seen within the next few days.

I: So, the explanation gives you some indication of how you should act?

P: Yes, but the action would lead into therapy or a therapy adjustment. And I think that adjustment would happen more through bringing the patient in, examining the patient clinically, speaking with the patient, and then initiating further diagnostics. So yes, this breakdown, together with the 83% probability, would motivate me to contact the patient.

7.4 SHAP

I: What information do you get from the explanation about the reliability or accuracy of the AI prediction? Could you infer any limitations or uncertainties of the AI model from it?

P: The limits and uncertainties simply come from the black box. You see these values and a probability is given, but you cannot understand it down to the last detail. If there were a certain probability, I would very likely bring the patient in for a clinical examination and then adjust my therapy based on that decision.

I: Would this graphical breakdown help you if the percentage prediction did not match your own clinical experience?

P: Yes, definitely. If I had time and was clicking through the patients and saw values that I considered concerning, for example an NT-proBNP of 10,000, but the system did not show an increased probability of decompensation, then I could understand why. Why is this value, which I normally associate with decompensation, not decisive enough for the system to say that the percentage is high? In that case, this exact graphic would make a lot of sense, because the other influencing factors would probably be broken down and explain why the result is not a high percentage.

I: Would the explanation help you recognize when you can trust the AI model's prediction and when you should be cautious?

P: Yes. But I think this is a process. At the beginning, you first have to trust the system. But if strange values come up, values that you cannot explain at all, or if the clinical course later shows that the system did not recognize a decompensation, then skepticism arises and you look more closely at the values. Still, this graphic you are showing me is really essential for understanding how the probability comes about and what influence the factors have. I think that is very important.

7.5 CFEs

I: To what extent does this table help you better understand and follow the prediction of the AI model?

P: It helps me understand, or try to understand, how the system thinks or wants to think. Of course, NT-proBNP is a very decisive value in assessing whether someone is decompensated or not. And above all, in the course of the disease, whether something is changing. You just said that these values

do not represent a trajectory but are only meant to explain how this probable decompensation assessment comes about. So, NT-proBNP certainly plays a very decisive role.

I: Which aspects of this tabular breakdown are particularly helpful or hindering for your understanding?

P: For me, this is plausible and it confirms my preconception that hemoglobin is a problem in this context, for whatever reason. But as a small criticism: if you present it like this, it is too many numbers for me. As someone who is no longer very young, I need reading glasses for this. The other visualization I can still read if necessary, but here you can also slip into the wrong line.

I: So, in terms of depth or level of detail, you would say it is rather too detailed or too complex?

P: Yes. If you really want to, you could hide it somewhere behind a click.

7.6 CFEs

I: So, if I understand correctly, you would prefer a more detailed explanation during the onboarding phase, using a few examples, and then visualizations that can be assessed at a glance?

P: Exactly. If you come with the software and explain at the beginning: these are the parameters we can analyze with the AI, and this is how the AI does it, this is how it will present it to you, and this is the background behind it, then that is okay during onboarding. But then I want to use it and quickly see and decide.

I: If we used this table as an example during onboarding, would it help you assess the reliability or accuracy of the AI prediction?

P: Yes, it could. To understand where the pitfalls of the AI are. If I know: okay, here the AI judged something incorrectly, and that is why it shows it to me like this. If I do not understand the AI at all and it tells me, "look at this patient," then it is useful to know why the AI might be so wrong. And if I have read through four, five, or six scenarios once, then I can say: ah, okay, this is how the thing works.

7.7 CFEs

I: Does this table give you an idea of how you should act in patient care?

P: Not more, no. We are back at the initial point. If you have a good cardiologist, they do not need this. If you have an assistant who has not been on duty for very long, it could make them think: maybe we have to consider how to raise hemoglobin. NT-proBNP does not help you at all, because you cannot influence it directly. It is only a marker of insufficiency. So, yes, this can help to understand things, but it can also lead in the wrong direction if it is misunderstood.

I: So, you would see this more as a theoretical model?

P: Yes, because you cannot just change such parameters ad hoc. Hemoglobin, for example, cannot just be raised like that. These are theoretical models. Whether and how that can be implemented in practice is another question.

7.8 Anchors

I: To what extent does this rule help you better understand the prediction of the AI model?

P: This is too under-complex for me, honestly. It leaves out a few points that I need. It tells me exactly what was not included. It does help, but for me there is a clear bias. The first visualization is much better. With little effort, it captures the amount of information I need to assess the situation.

I: So, you would say this provides too little information?

P: Yes.

I: Does this help you draw any conclusions about whether there is a need for action or whether it can be used meaningfully in everyday work?

P: No. I can see that above at a glance. The first visualization was the most plausible to me because it includes all parameters in one simple view. The second one makes me look at too many numbers, and this one is much too little. I already knew that high NT-proBNP and low hemoglobin are not good. You learn that fairly early.
